# The Impact of a Digital Artificial Intelligence System on the Monitoring and Self-management of Nonmotor Symptoms in People With Parkinson Disease: Proposal for a Phase 1 Implementation Study

**DOI:** 10.2196/40317

**Published:** 2022-09-26

**Authors:** Edward Meinert, Madison Milne-Ives, K Ray Chaudhuri, Tracey Harding, John Whipps, Susan Whipps, Camille Carroll

**Affiliations:** 1 Centre for Health Technology University of Plymouth Plymouth United Kingdom; 2 Department of Primary Care and Public Health School of Public Health Imperial College London London United Kingdom; 3 Harvard TH Chan School of Public Health Harvard University Boston, MA United States; 4 Department of Basic and Clinical Neuroscience The Maurice Wohl Clinical Neuroscience Institute King’s College London London United Kingdom; 5 School of Nursing and Midwifery Faculty of Health University of Plymouth Plymouth United Kingdom; 6 University of Plymouth Plymouth United Kingdom; 7 Peninsula Medical School Faculty of Health University of Plymouth Plymouth United Kingdom

**Keywords:** Parkinson disease, self-management, telemedicine, artificial intelligence

## Abstract

**Background:**

Nonmotor symptoms of Parkinson disease are a major factor of disease burden but are often underreported in clinical appointments. A digital tool has been developed to support the monitoring and management of nonmotor symptoms.

**Objective:**

The aim of this study is to establish evidence of the impact of the system on patient confidence, knowledge, and skills for self-management of nonmotor symptoms, symptom burden, and quality of life of people with Parkinson and their care partners. It will also evaluate the usability, acceptability, and potential for adoption of the system for people with Parkinson, care partners, and health care professionals.

**Methods:**

A mixed methods implementation and feasibility study based on the nonadoption, abandonment, scale-up, spread, and sustainability framework will be conducted with 60 person with Parkinson–care partner dyads and their associated health care professionals. Participants will be recruited from outpatient clinics at the University Hospitals Plymouth NHS Trust Parkinson service. The primary outcome, patient activation, will be measured over the 12-month intervention period; secondary outcomes include the system’s impact on health and well-being outcomes, safety, usability, acceptability, engagement, and costs. Semistructured interviews with a subset of participants will gather a more in-depth understanding of user perspectives and experiences with the system. Repeated measures analysis of variance will analyze change over time and thematic analysis will be conducted on qualitative data. The study was peer reviewed by the Parkinson’s UK Non-Drug Approaches grant board and is pending ethical approval.

**Results:**

The study won funding in August 2021; data collection is expected to begin in December 2022.

**Conclusions:**

The study’s success criteria will be affirming evidence regarding the system’s feasibility, usability and acceptability, no serious safety risks identified, and an observed positive impact on patient activation. Results will be disseminated in academic peer-reviewed journals and in platforms and formats that are accessible to the general public, guided by patient and public collaborators.

**Trial Registration:**

ClinicalTrials.gov NCT05414071; https://clinicaltrials.gov/ct2/show/NCT05414071

**International Registered Report Identifier (IRRID):**

PRR1-10.2196/40317

## Introduction

### Background

The aging population of the United Kingdom is expected to nearly double the prevalence of Parkinson disease by 2065 [1]. Parkinson disease significantly impacts patients, their care partners and families, and the health system of the United Kingdom. However, people with Parkinson disease are not monitored continuously or even frequently [2]; the National Institute for Health and Care Excellence guidelines currently recommend that people with Parkinson disease be reviewed every 6 to 12 months [3]. Nonmotor symptoms (NMS) of Parkinson can increase disease burden and decrease quality of life [4,5] but are often overlooked or undeclared in routine appointments [6]. Digital technology has the potential to improve the identification of NMS and enable more timely and appropriate treatment.

In this study, we will build on this evidence to implement improvements to a digital self-management system for nonmotor symptoms (NMS Assist) and to examine the impact of NMS Assist on patient activation and remote NMS monitoring (specifically the health care contacts triggered by that monitoring using NMS Assist). The high proportion of the UK population with internet access [7] means that this intervention could easily be made available on a large scale to people with Parkinson disease and their care partners.

### Rationale

#### Benefits for People With Parkinson Disease and Care Partners

NMS Assist is expected to have several benefits for people with Parkinson disease and care partners; notably, by improving symptom monitoring by making assessments more timely, relevant, and convenient, by increasing disease and symptom awareness, and by supporting people with Parkinson disease and care partners in managing their NMS at home. A formative usability evaluation of 13 users (9 people with Parkinson disease, 4 care partners) demonstrated satisfaction with the system (System Usability Score 80%) [8].

The usability study identified 11 critical issues for improvement, centered on 3 themes: navigation, content, and accessibility [8]. These aspects of the system were subsequently refined based on participants’ suggestions. To reduce the length of the NMSQ, people with Parkinson disease can complete partial assessments to monitor their symptoms. However, they are asked to complete the length of the Nonmotor Symptoms Questionnaire (NMSQ) every 6 months to ensure that a holistic assessment is performed, providing clinical safety netting.

#### Benefits for Health Care Professionals

NMS Assist will identify unmet need among people with Parkinson disease, and the adoption of a new system will add an element to the workload of health care professionals (HCPs); new skills will be needed to make clinical decisions based on the data developed. However, NMS Assist will provide HCPs with the ability to monitor and prioritize their patients efficiently. The main long-term benefit is the development of shared responsibility of health management with people with Parkinson disease, achieving the ambitions of the National Health Service (NHS) Long Term Plan with digitally enabled care and delivery of predictive, personalized, preventive and participatory care medicine, with improved support for care partners [9]. In time, this may result in fewer NMS-related complications (eg, falls), hospitalizations, and institutionalized care.

#### Benefits for Parkinson Disease Research

Ongoing collection of data will enable a longitudinal database. This will enable evaluations to identify clusters of NMS and associations between NMS and people with Parkinson disease and care partner characteristics. The value of this database for research would only be increased if NMS Assist was adapted and adopted in countries worldwide. For instance, it would enable examining global similarities or differences in symptoms and symptom clusters, quality of life impairment, and impact on care partners.

### Theoretical Frameworks

The nonadoption, abandonment, scale-up, spread, and sustainability (NASSS) framework [10] that aims to help predict and evaluate the impact of digitally enabled health programs was used to support the conception and development of the study plan. It was chosen as the main theoretical framework to ensure that the evaluation would examine a holistic set of variables essential to the long-term success and sustainability of the NMS Assist system. Qualitative data will be collected through semistructured interviews with a subset of users structured according to the theoretical framework of acceptability [11]. The use of both quantitative and qualitative methods to evaluate usability will enable the data to be triangulated.

These 2 frameworks will form the theoretical basis for the evaluation of NMS Assist:

- Long-term adoption and suitability to further trials will be evaluated using the NASSS framework [10]. This framework is important to include because interventions can only add value if they are successfully adopted and used. This framework emphasizes the consideration of the multiple levels—individuals and systems—that influence adoption and nonadoption.
- The theoretical framework of acceptability will be used to structure the semistructured interview guide [11]. This framework was chosen because it was developed specifically for health interventions, captures a multifaceted view of acceptability (with 7 components), and emphasizes the importance of considering different time points when evaluating acceptability [11].

### Research Question

The main research question for the study is as follows: How does a digital remote monitoring and education system (NMS Assist) impact patient activation, symptoms, and quality of life for people with Parkinson disease and care partners?

### Aims and Objectives

The study will assess the impact of NMS Assist on people with Parkinson disease, their care partners, and their Parkinson care team. Evidence regarding the system’s perceived impact on knowledge, confidence, and skills at managing NMS and its safety, acceptability, and usability will improve the product design and determine readiness for advancement for wider adoption.

To achieve this aim, there are 5 key objectives:

- Assess the impact of the NMS Assist system on patient activation, symptom burden, and quality of life in people with Parkinson and their care partners
- Examine the impact of the NMS Assist system on contact between people with Parkinson disease and HCPs
- Assess the acceptability and usability of the system and identify any barriers to use for people with Parkinson, their care partners, and health care professionals
- Assess the cost impacts of the system compared to the standard of care
- Establish whether there is sufficient evidence of the impact of NMS Assist on the knowledge, confidence, and skills of people with Parkinson disease managing NMS, the identification of new or problematic NMS, and the prompting of health care contacts to justify more extensive trials

Based on evidence gathered from previous studies and our understanding of state of the art concerning digital health technologies and ongoing work with digital tools used with people with Parkinson disease, we hypothesize the following:

- NMS Assist will be acceptable, accessible, and engaging for users
- Use of NMS Assist will be feasible within an NHS service
- NMS resources will have a positive impact on patient activation (knowledge, skills, and confidence)

## Methods

### Study Design

**Figure 1:**
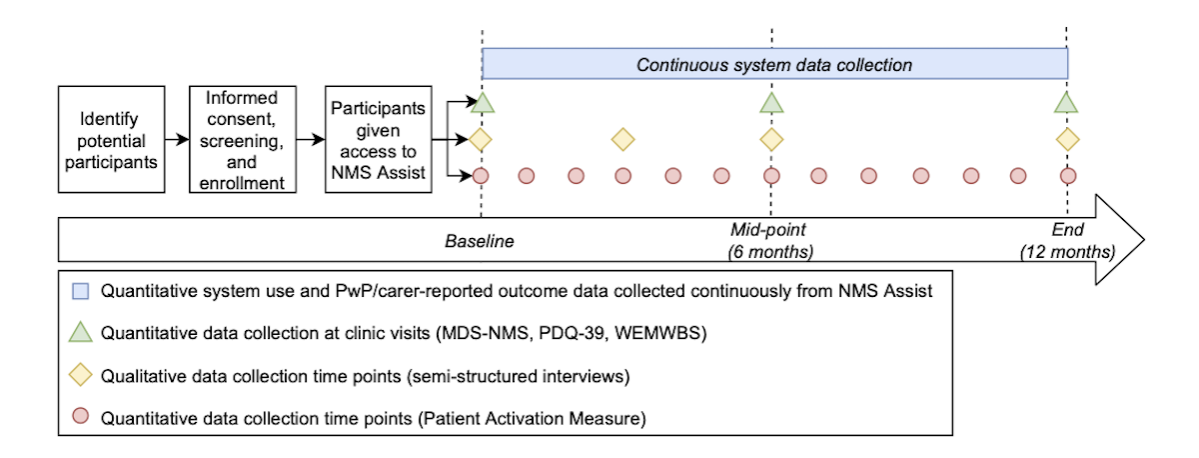
Study sequence diagram. NMS: Nonmotor symptoms. PwP: People with Parkinson disease. MDS-NMS: Movement Disorder Society–Nonmotor Rating Scale. PDQ-39: Parkinson’s Disease Questionnaire–39. WEMWBS: Warwick-Edinburgh Mental Wellbeing Scale.

This investigation will be a mixed methods feasibility study using an implementation science theoretical framework [12] to examine the research question. Quantitative methods will be used to evaluate NMS Assist’s impact on patient activation as well as to examine health and well-being outcomes, usability, and engagement. Qualitative methods, specifically semistructured interviews, will also be used to gather a more in-depth understanding of the acceptability, usability, and engagement of users with NMS Assist (see Figure 1 for an overview). The SPIRIT (Standard Protocol Items: Recommendations for Interventional Trials) checklist was used to ensure the proposal was meeting content and quality standards (Multimedia Appendix 1) [13].

### Intervention

**Figure 2:**
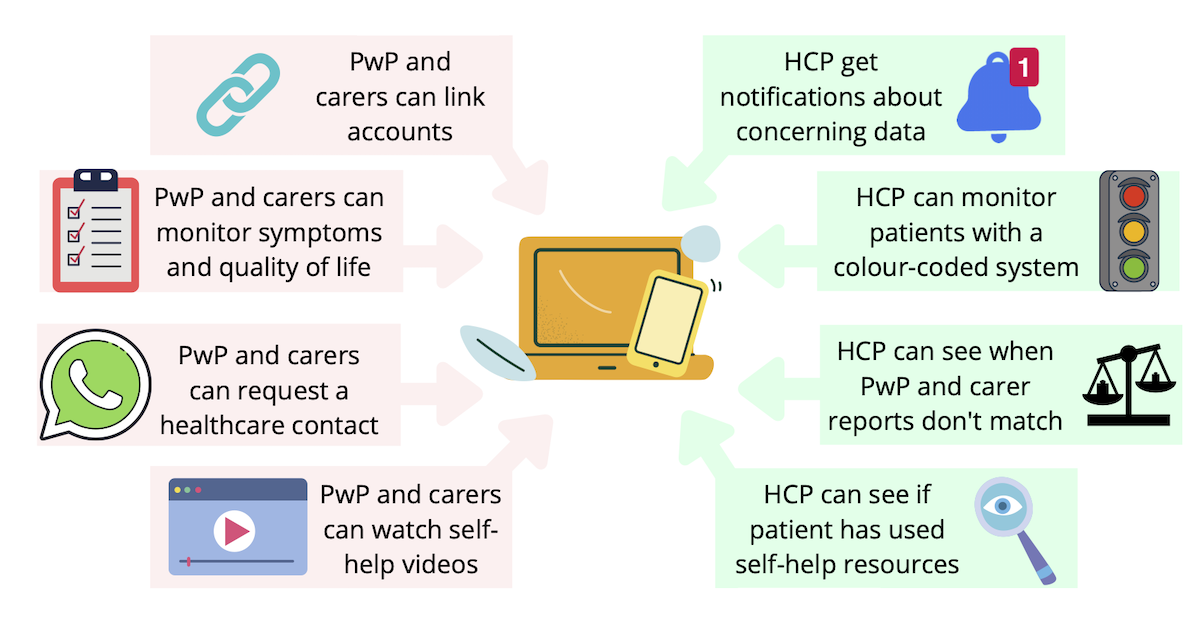
NMS Assist system features. PwP: People with Parkinson disease. NMS: Nonmotor symptoms. HCP: health care professionals.

NMS Assist is a patient engagement system that comprises a front-end patient engagement app and a back-end clinical data management system. Patient engagement systems can communicate with patients to collect data, provide feedback and educational resources, and support the management of a patient-provider relationship. For all Parkinson symptoms, there are pharmacological and nonpharmacological approaches to management. As motor symptoms are already well-supported by digital health solutions, NMS Assist focuses on the nonpharmacological approaches to nonmotor symptoms. It provides initial support for people with Parkinson disease and care partners to identify and manage their NMS before they can be seen by an HCP or need medication. The system’s self-help resources go through the first 3 steps that an HCP would explore with a patient: (1) identifying and describing the symptom using validated questionnaires (Nonmotor Symptoms Questionnaire [NMSQ] [14], Parkinson’s Disease Questionnaire–8 [PDQ-8] [15], and Parkinson’s Disease Questionnaire for Carers [PDQ-C] [16]), (2) explaining why it happens in Parkinson, and (3) providing nonpharmacological approaches to managing it. The system then has the ability to request health care contact for people with Parkinson disease who are still struggling to manage their symptoms so that the HCP can explore further measures. Steps 2 and 3 are supported by self-help videos that use diverse characters and were designed to ensure visual discrimination and easily translatable captions to address visual, hearing, and motor difficulties. The app is also designed to allow care partners to record and monitor their well-being and how they feel the person with Parkinson disease is getting on (see Figure 2). The intervention will be delivered in addition to usual care to the person with Parkinson disease.

### Sample Selection and Recruitment

People with Parkinson disease attending outpatient clinics through the University Hospitals Plymouth NHS Trust Parkinson service are eligible for this study (see Textbox 1 for eligibility criteria). All people with Parkinson disease and care partners who are willing and eligible will be included, with the intention of recruiting a representative sample based on various demographic and Parkinson-specific characteristics. The participants invited to the semistructured interview will be randomly selected from within different demographic and disease characteristic categories; we will continue recruitment in the semistructured interviews until we have reached demographic saturation to ensure we complete in-depth qualitative understanding of the impact of the system.

Recruitment will take place through the Plymouth Parkinson service. People with Parkinson disease attending outpatient clinics at the Plymouth Parkinson service will be invited to participate in the study along with their care partner. The associated HCPs responsible for the care of people with Parkinson disease and care partner dyads who consent into the study will then be invited to participate as well. Patients will be recruited during clinical appointments with the Plymouth Parkinson service by research nurses who will provide them with a paper participant information sheet to consider and will collect verbal or written consent using a preestablished informed consent form. Associated HCPs of the people with Parkinson disease who consent into the study will be invited to participate in the study for use of the clinician portal (and provided with an HCP version of the participant information sheet and informed consent form).

**Selection criteria. NMS: Nonmotor symptoms.**

Inclusion criteria:

- Willing and able to provide informed consent and comply with intervention requirement
- Aged 18 years or older
- Be fluent in English
- Not resident in a care or nursing home
- Ambulatory
- Have compatible smartphone and data access
- Normally under the care of the Parkinson service in the participating organization
- Participant’s health care professional in the participating organization consented to participate in the study

Exclusion criteria:

- Incapable of self-consent
- Secondary cause of parkinsonism
- Significant cognitive impairment or a diagnosis of Parkinson disease dementia
- Living in residential care facilities
- Previous involvement in development or testing of the NMS Assist system
- A life expectancy of <6 months
- Significant comorbidity, which in the opinion of the chief investigator would preclude safe participation in the study or protocol compliance
- In a dependent or unequal relationship with the research or care teams or any patient and public involvement representatives

### Sample Size

We will recruit 60 people with Parkinson disease–care partner dyads and their associated HCPs—the exact number of HCPs cannot be determined until the people with Parkinson disease–care partner dyads have been recruited, as we will include HCPs who are associated with those patients. Of the 60 dyads, at least 20 people with Parkinson disease–care partner dyads will be purposively sampled for a semistructured interview. We will also be inviting the HCPs associated with those dyads to separate semistructured interviews.

Since this is a feasibility study, a formal sample size calculation has not been performed. A sample size of 60 people with Parkinson disease–care partner dyads was selected because this is an achievable number of patients to recruit within the study period based on our prior research.

### Outcomes

#### Primary Outcome

The primary outcome, patient activation, will be assessed using the Patient Activation Measure [17]. The concept of patient activation refers to patients having the necessary knowledge, confidence, and skills to manage their own health and well-being. It is important to note that as this study design is centered on feasibility, the evidence captured will not measure efficacy. Should there be indications of a positive impact of the NMS Assist system on patient activation and health and well-being outcomes, efficacy will be tested in further studies.

#### Secondary Outcomes

There will be several secondary outcome measures to assess health and well-being outcomes, safety, feasibility, usability, and acceptability (see Textbox 2).

**Secondary outcomes and measures. EQ-5D-5L: EuroQol 5 Dimension 5 Level. NMS: Nonmotor symptoms.**

1. Health outcomes

- Nonmotor Symptoms Questionnaire [14]
- Movement Disorder Society–Nonmotor Rating Scale [18]
- Parkinson’s Disease Questionnaire–8 [15]
- Parkinson’s Disease Questionnaire–39 [19]
- EQ-5D-5L [20]

2. Well-being outcomes

- Warwick-Edinburgh Mental Wellbeing Scale [21]
- Parkinson Disease Questionnaire for Carers [16]

3. Safety

- Aggregated evaluation results
- Adverse event reporting

4. Healthcare contacts

- Number of patient-initiated contacts with health care team (through the ‘request health care contact’ feature)
- Number of health care professional–initiated contacts (NMS Assist system has identified a need for clinical intervention based on patient- or care partner–reported data and prompted the health care professional to contact patient) recorded by the system

5. Feasibility

- Technical issues identified from the system use data
- Technical issues identified by participants

6. Usability

- System Usability Scale [22,23]
- Qualitative feedback from semistructured interviews

7. Acceptability

- Qualitative feedback from semistructured interviews (structured using the theoretical framework of acceptability) [11]

8. Engagement

- System use data (trend of use over time)
- Qualitative feedback from semistructured interviews

9. Costs

- Cost analysis will be used to examine the factors impacting costs for implementing the system

##### Health and Well-being Outcomes

A key element of the NMS Assist system is the use of validated questionnaires, and several of the health and well-being outcomes will be measured through the app—including the NMSQ [14], the short form of the PDQ-8 [15], and the PDQ-C [16]. The questionnaires have been validated in several different populations (eg, NMSQ [14,24-26], PDQ-8 [27-31]), suggesting that the measurement properties are appropriate for people with Parkinson disease in different contexts [32].

During the clinic visits (at baseline, 6 months, and 12 months), health and well-being outcomes will be assessed using the long-form PDQ-39 [19], the Warwick-Edinburgh Mental Wellbeing Scale (WEMWBS) [21], the Movement Disorder Society–Nonmotor Rating Scale (MDS-NMS) [18], and the EQ-5D-5L (EuroQol 5 Dimension 5 Level) [20]. These validated measures will be used to gather more in-depth data about people with Parkinson disease symptoms and their impact on people with Parkinson disease and care partner well-being to provide additional evidence of the impact of the system on health and well-being. This will also enable an assessment of the validity of home-collected measures.

##### Safety

We do not anticipate any significant safety risks from the intervention as the self-help guidance was collected from credible resources, but safety will be assessed by maintaining an adverse event report log.

##### Health Care Contacts

The use of the health care contact function will be assessed by examining the number and frequency of people with Parkinson disease– and HCP-initiated contacts through the system.

##### Feasibility

Feasibility will be assessed by examining any technical issues identified in the system and system use data (including logs of complaints made by participants during the intervention delivery period) as well as asking participants about any technical issues they encountered when using NMS Assist during the semistructured interviews.

##### Usability

Usability will be assessed using questions presented to people with Parkinson disease, care partners, and HCPs delivered by online or paper surveys distributed to participants during their clinical visits at the beginning, midpoint, and end of the study delivery period. These questions will be drawn from the System Usability Scale [22]. Usability will also be assessed during the qualitative semistructured interviews by asking about any usability issues or barriers (not due to technical malfunctions) encountered by the users.

##### Acceptability

The acceptability of NMS Assist will be assessed during qualitative interviews with a subset of the people with Parkinson disease, care partners, and HCPs. These semistructured interviews will enable participants to provide more in-depth feedback about what they liked about NMS Assist and any barriers or frustrations they experienced using the system. The theoretical framework of acceptability will be used to structure the questions asked about acceptability in the semistructured interview guide because it considers several different facets of acceptability before, during, and after the intervention delivery [11]. Perceived acceptability at these different time points is likely to have an impact on the adoption and sustained engagement with NMS Assist.

##### Engagement

System use data will be collected to gather data about participants’ engagement with the NMS Assist system. However, system use data only captures one component of engagement and is not a valid measure of engagement on its own [33,34]. Engagement has cognitive, behavioral, and affective aspects [34-37], and engagement with a digital health intervention includes engagement with the system itself, with various components of the intervention, and with the health behaviors it is trying to support. Therefore, system use data will be supplemented with semistructured interviews and a user engagement questionnaire (eg, the eHealth Engagement Scale [38] or the User Engagement Scale [39]) to gain a better understanding of user experience engaging with the intervention and the recommended behaviors.

##### Costs

A cost analysis impact of implementing the NMS Assist system will be conducted. Resource use (time spent on the system) and costs to implement throughout the 12-month intervention period to be compared to published data on costs for the standard of care.

### Study Duration and Follow-up

The study will consist of 3 main phases over 24 months: preparation (6 months), delivery of the intervention (12 months, see Figure 1 for follow-up data collection points), and analysis and dissemination (6 months). The study team will monitor the use of NMS Assist and assure the system is being used within designed parameters during the study. No incentives will be provided to encourage adherence; the study is assessing the use of NMS Assist in the real world, and we expect that the benefits for people with Parkinson disease and care partners provided by the monitoring and self-help features will sufficiently encourage adherence to the study.

### Data Collection

The evaluation shall follow the NASSS framework [10] that aims to help predict and evaluate the impact of digitally enabled health programs (see Table 1).

**Table 1:** Description of quantitative and qualitative data collection strategies structured using the nonadoption, abandonment and challenges to the scale-up, spread, and sustainability framework [10].

NASSS^a^ domain and participants		Quantitative data	Qualitative data (topics in SSI^b^)
**Illness or condition**			
	All	Participant characteristics (eg, age, sex, cognitive impairment, depression, digital literacy)	Appropriateness of the system for individual context
	People with Parkinson disease and care partners	Disease characteristics	—^c^
**Technology**			
	All	Usability	Usability (barriers and facilitators to use)
	All	—	Perceptions of system features, data privacy, and security
	HCP^d^	System use data	Perceptions of the data provided by the system and use of data to improve understanding or inform care decisions
	People with Parkinson disease and care partners	System use data	Perceptions of self-management resources and system feedback
**Value proposition**			
	All	—	Motivation to participate in the study
	All	—	Suggestions for system improvement
	People with Parkinson disease and care partners	Care partner quality of life	Perceptions of impact of system on confidence, knowledge, and skills for managing nonmotor symptoms.
	People with Parkinson disease and care partners	Nonmotor symptoms and their burden	Perceptions of impact of system on symptom burden
	People with Parkinson disease and care partners	Patient functioning and well-being	Perceptions of impact of system on quality of life
	People with Parkinson disease and care partners	Patient’s knowledge, confidence, and skills (patient activation) at managing their health care	—
	—	Costs and time commitments will be evaluated as part of the cost assessment using quality of life data collected in the study and published data on existing care treatment costs	—
**Adopter system**			
	All	—	Acceptability
	All	—	Barriers and facilitators to adoption
	HCP	Characteristics of high and low system users	Reasons for high or low engagement with system
	People with Parkinson disease and care partners	Proportion who completed questionnaires and frequency of completion	Effort required to learn and use the system
	People with Parkinson disease and care partners	Proportion who accessed the self-help resources relevant to their specific symptoms (as reported on the NMSQ^e^)	Perceived impact of system on care partner burden
**Organization**			
	HCP	System use data (eg, number of log-ins per user, time spent on system, number of notifications responded to or actioned)	Issues reported with implementing the system in care practices
	HCP	—	Perceptions of impact of system on routine care processes, workflows, and workloads
**Wider context**			
	—	Implications for the national standards of care and inclusion in preexisting care pathways will be investigated	—
**Embedding and adaptation over time**			
	All	Participant retention throughout the study period	Reasons for continued/discontinued use of system during study
	All	System use data (engagement) throughout study period	Perspectives on continued use and adaptations of the system in future
	—	Costs and time commitments will be evaluated as part of the cost assessment using quality of life data collected in the study and published data on existing care treatment costs	—

^a^NASSS: nonadoption, abandonment and challenges to the scale-up, spread, and sustainability.

^b^SSI: semistructured interview.

^c^Not applicable.

^d^HCP: health care professional.

^e^NMSQ: Nonmotor Symptoms Questionnaire.

#### Quantitative Data Collection

The primary outcome, patient and care partner activation, will be completed at the beginning of the intervention period and monthly thereafter to measure the impact of NMS Assist on the knowledge, confidence, and skills of people with Parkinson disease and care partners over time [17] (see Figure 1). People with Parkinson disease and care partner scores on the NMSQ, PDQ-8, and PDQ-C will be recorded automatically by the system. The proportion of people with Parkinson disease who are contacted by their nurse or clinician based on their symptom questionnaire responses will be measured. Quantitative app and portal use data will be captured by the system to examine patterns of engagement with the questionnaires, self-help resources, and triggered health care contacts. Data about participant characteristics, recruitment, and retention will also be recorded.

Participants will also be asked to attend 3 study visits, at baseline, 6 months, and 12 months (see Figure 1), where they will complete the long-form PDQ-39 [19] and the Warwick-Edinburgh Mental Wellbeing Scale (WEMWBS) [21] and will have administered the Movement Disorder Society–Nonmotor Rating Scale (MDS-NMS) [18] and the EQ-5D-5L [20].

#### Qualitative Data Collection

Qualitative data will be collected at 4 time points: baseline, 3, 6, and 12 months (see Figure 1). Purposive sampling will identify 20 participants, including people with Parkinson disease (with various demographic and disease characteristics), care partners, and HCPs, for qualitative semistructured interviews. The interviews will assess the system’s acceptability structured using the theoretical framework of acceptability [11]. They will also explore participant motivation to participate, their engagement with the system, and any barriers or facilitators to use, usability issues, or technical difficulties they encounter. The perceived impact of the system on the knowledge, confidence, skills, and symptom burden of people with Parkinson disease and care partners will also be investigated to triangulate with the quantitative measures.

### Data Analysis

The primary analysis will be the comparison of mean Patient Activation Measure scores [17] at each month. A repeated measures analysis of variance will be conducted to identify any significant changes in mean Patient Activation Measure scores over the 13 time points (baseline and monthly until 12-months postbaseline). If the normality assumption for a repeated measures analysis of variance is not met, a nonparametric alternative will be used.

The same analysis for the primary outcome will be conducted for the health and well-being outcome measures and the System Usability Scale, comparing the measure scores at the baseline, midpoint, and end points. Exploratory analyses will be conducted to identify potential associations between demographic or disease characteristics and outcome measures to inform hypotheses for future evaluations. All other quantitative data will be analyzed using descriptive statistics.

Semistructured interviews will be coded by 2 investigators using thematic analysis [40]. Triangulation of the quantitative questionnaire, system use, and qualitative semistructured interview data will be conducted to validate findings.

### Ethical Considerations

#### Ethics Approval

Ethical approval is being sought from the Health Research Authority and the relevant Research Ethics Committee [41]. The study is registered at ClinicalTrials.gov [NCT05414071].

#### Confidentiality, Data Management, and Consent

The NMS Assist system will store identifiable data as part of the clinical record. The solution is in compliance with the UK Data Protection Act 2018 [42]. Each participant will be given a unique identifier with the key stored securely for reference purposes. Only the researchers will have access to research data and only the HCPs will have access to their patients’ clinical data. Interview recordings will be anonymized and destroyed following transcription. After having the opportunity to review the participant information sheet, which explains the study procedures and provides clear information (reviewed by the people with Parkinson disease and care partner coapplicants) about confidentiality, data privacy, and security and participant rights, participants will be asked to provide explicit consent for specific aspects of the study and data management.

#### Recruitment

Participant eligibility will be determined by the screening process they undergo in outpatient care at the University Hospitals Plymouth NHS Trust. All eligible participants will be invited to participate with no influence from the researchers. Interview participants will also be randomly selected (from demographic groups) by a computer script of intervention users. All participants will be included in the process evaluation’s quantitative analyses, thus avoiding recruitment bias.

#### Power Relations and Other Potential Biases

Preestablished inclusion and exclusion criteria will be used to avoid any potential bias from the researchers as well as any coercion from participants. To prevent participation bias, only participants who would not otherwise progress down the standard care pathway will be excluded.

#### Sensitivity

The outcomes measures that concern potentially sensitive topics (personal health and well-being) will be delivered to the patients’ phones via the app or by their HCP in a standard clinical setting. This enables them to avoid discussing any sensitive topics with a researcher. The semistructured interview questions will focus only on the participants’ experiences with the app (engagement and acceptability) and are intended to avoid any areas of cultural or psychological sensitivity.

#### Timing

To mitigate any time concerns for participants, qualitative interviews will last for a maximum of 60 minutes. If this is a concern for any of the people with Parkinson disease, the interviews can take place over 2 sessions.

#### Study Governance

A project management group comprising the chief investigator, coinvestigators (including patient and public involvement [PPI] coinvestigators), an external researcher, and additional PPI representatives from the Community for Research Involvement and Support by People With Parkinson Diseases (King’s College London) will review progress to provide study governance and oversight and to ensure there are no ethical concerns with the study. Planned deviations or waivers to the protocol are not allowed under the UK regulations on clinical trials and will not be used.

### Safety Considerations

A comprehensive risk register and hazard log has been maintained for NMS Assist across its development as part of its clinical safety management file. This risk register will be regularly reviewed and updated as part of the project management governance. Key risks for this project include the following:

- Clinical: people with Parkinson disease, care partners, or HCPs are unwilling to incorporate the system into routine use; mitigated by a prior extensive cocreation process focused on user-centered design
- Excessive screen time: health risks associated with excessive screen time are mitigated encouraging users to complete the strategies and skills taught by the app away from the screen. All risks and mitigations are outlined in the clinical safety management file
- Technical: the system provides inappropriate decision support for clinicians; mitigated by supervision by the clinical research team, review of any errors, and extensive safety evaluation

## Results

The study was awarded funding for 24 months in August 2021. Data collection is expected to begin in December 2022 and the study is expected to be completed by March 2024.

## Discussion

### Anticipated Findings

The evaluation is expected to show that the NMS Assist system has a positive impact on patient and care partner knowledge, skills, and confidence at managing NMS and general well-being. In addition, the results are expected to demonstrate the acceptability and accessibility of the system for users and its feasibility for implementation within NHS services.

### Strengths and Limitations

A key strength of the intervention and study protocol is their co-design and development with PPI coinvestigators. The full execution of the study will be conducted in collaboration with PPI coinvestigators and other PPI representatives. Another strength is that the study has a long follow-up period (12 months) over which participants can use the NMS Assist system, which will enable an examination of the sustainability of the system’s adoption in clinical and home settings.

A limitation of the study is its ability to assess the health and well-being outcomes measured. The study design does not enable a comparison between groups, which would provide stronger evidence of the system’s potential impact. The generalizability of the results is also limited by the system, which requires that participants own a compatible device and be fluent in English.

### Dissemination Plan

Analysis of the data will be compiled into a published peer-reviewed paper. People with Parkinson disease, care partners, and HCP investigators involved in the project will be integral in disseminating the results. PPI representatives will help develop and edit communications for all audiences, but, in particular, the summary of results that will be sent to all study participants. They will take the lead in disseminating and presenting results to the broader Parkinson community and professional groups such as through patient networks, the Parkinson’s UK research network, patient and care partner congresses and conferences and the World Parkinson’s Congress (which includes patients and professionals), newsletters, articles, and social media. Academic members of the project team will disseminate the results through peer-reviewed journal publications and at research conferences.

The newest version of NMS Assist will be made publicly available to people with Parkinson disease and care partners after the system refinement stage (6 months after project commencement). This access will be available concurrent to our study, and members of the public will have the ability to consent into the study if they choose. We will make the data collected by NMS Assist freely accessible for research purposes by creating a data set that can be stored and shared securely in a data safe haven. To ensure that this data asset is used most appropriately, we will consult with Parkinson’s UK and other stakeholders when developing our data sharing policy. In future, this will enable us to provide free and secure access to the data for people who apply to use it for research purposes, while accounting for data security and ethical considerations.

## Appendix

Multimedia Appendix 1 Standard Protocol Items: Recommendations for Interventional Trials checklist.

Multimedia Appendix 2 Peer-review report (stage 1) from the Parkinson’s UK - Grants for Non-Drug Approaches (London, UK).

Multimedia Appendix 3 Peer-review report (stage 2) from the Parkinson’s UK - Grants for Non-Drug Approaches (London, UK).

## Abbreviations

EQ-5D-5L: EuroQol 5 Dimension 5 Level
HCP: health care professional
MDS-NMS: Movement Disorder Society–Nonmotor Rating Scale
NASSS: nonadoption, abandonment, and challenges to the scale-up, spread, and sustainability
NHS: National Health Service
NMS: nonmotor symptoms
NMSQ: Nonmotor Symptoms Questionnaire
PDQ: Parkinson’s Disease Questionnaire
PPI: patient and public involvement
SPIRIT: Standard Protocol Items: Recommendations for Interventional Trials
WEMWBS: Warwick-Edinburgh Mental Wellbeing Scale

## Data Availability

Data will be available on request from the corresponding author but will not be publicly available due to them containing information that could compromise participant privacy or consent.
