# Supplementary material for: The Impact of a Digital Artificial Intelligence System on the Monitoring and Self-management of Nonmotor Symptoms in People With Parkinson Disease: Proposal for a Phase 1 Implementation Study
Source: JMIR Res Protoc. 2022 Sep 26;11(9):e40317. doi: 10.2196/40317 (PMC9555326; doi:10.2196/40317)
Supplement: Multimedia Appendix 2 [file resprot_v11i9e40317_app2.pdf]

College of Experts
Non drug approaches grant pre-proposal reviewer form

PARKINSON'S UK
CHANGE ATTITUDES.
FIND A CURE.
JOIN US.

This form should be completed by the College of Expert reviewer for a non drug approaches grant pre-proposal. The information you provide is maintained as a record of the decision-making process and to give feedback to applicants.

If you have any questions, please contact the Research team by emailing researchapplications@parkinsons.org.uk.

Application reference (Internal use)	NDA-21-15	Reviewer reference (Internal use)	
Principal applicant	Dr Edward Meinert<br>Dr Camille Carroll		
Application title	The impact of a digital portal on the monitoring and self-management of nonmotor symptoms in people with Parkinson’s		

Please do remember to provide a score for your evaluation at the end of this review.

Importance

- How important are the questions, or gaps in knowledge, that are being addressed? Is there a good rationale for pursuing these? Is success likely to lead to significant new understanding?
- Is the research relevant to Parkinson's?
- Does the proposal realistically set out the ultimate potential health or social gain to people with Parkinson's?
- How important is it to do the work now?
- Is there similar or complementary research underway elsewhere? Are the proposals competitive?

The application provides a strong case for the need for this research, highlighting the anticipated increase in prevalence of Parkinson’s by 2016, and highlighting how non-motor symptoms in particular are a troubling, yet often overlooked, aspect of the condition. Coupled with the ongoing COVID-19 pandemic, an investigation into a novel method of remote NMS monitoring to improve clinical intervention and quality of life is more important than ever.

I do believe the application would benefit from further insight into the novelty of the NMS Assist application – what novelty does it provide over similar approaches?

Environment and people

- Have the applicants established a high-quality track record in the field, including publication history?
- Are the applicants uniquely placed to deliver the work?
- Where the proposal embarks on work in a field new to the applicants, or is a first funding application, is there a firm foundation to take the work forward?
- How well does the work fit with other relevant research pursued by the applicants?

Members of the research team are well placed to deliver this work, possessing a good balance of technical and clinical expertise, in addition to representation of people with Parkinson’s and their caregivers. Both lead applicants have an impressive track record of research grants and publications in relevant areas.

Parkinson’s UK is the operating name of the Parkinson’s Disease Society of the United Kingdom. A company limited by guarantee. Registered in England and Wales (948776). Registered office: 215 Vauxhall Bridge Road, London SW1V 1EJ. A charity registered in England and Wales (258197) and in Scotland (SC037554).

It is particularly interesting to see that Dr Meineart has interests in clinical artificial intelligence – will this be integrated into NMS Assist? I believe this could be an excellent source of novelty.

Research plans

- Will the proposed plan of work achieve the objectives outlined in the proposal?
- Are the methodological plans realistic, given the aims of the research and the resources?
- Are the methods and study designs appropriate and free of flaws?

I believe the research team have the ability to achieve the proposed objectives within the time frame outlined in the proposal. The applicants have allocated contingency time to areas which are often prone to delay, such as ethics approval. The methods and study design appear to be well thought out.

It would be beneficial to provide more detail on planned recruitment. Will you recruit participants from a wide range of ages, with a wide range of symptoms and at various stages of their Parkinson's journey? The stratification of participants may provide interesting insight into who can most benefit from this intervention.

The application mentions that responses to remote monitoring that trigger additional service support will be measured – further details of this process would be beneficial.

Justification of resources requested

- Is the number of staff appropriate for the work described? Are the reasons for purchasing major items of equipment clearly set out?
- Are the funds requested appropriate for the work proposed and does the scientific potential justify funding on the scale requested? Is the project value for money?
- Are the timescale and scheduling realistic?

As a significant amount of funding (£50,000) will be dedicated to the subcontracting of software development on this already implemented solution, it would be beneficial for the applicants to provide some further detail of the types of additional development that are envisioned. It would also be beneficial to show when this software development is anticipated to take place within Appendix A (Gantt Chart)

Evidence of potential impact and clear pathway for benefit to people with Parkinson's in the near future

With these awards we are focused on delivering outcomes that make a difference to people with the condition. That means that we're looking for projects that, if successful, have a clear pathway to be made available to people affected by Parkinson's.

Please let us know whether this is apparent in this pre proposal application

This work is of clear importance, however, I do feel the application would benefit from further insight into the novelty of this work, and evidence-based potential benefits to quality of life / clinical intervention / costs that may result. Additionally, further insight into anticipated pathway to clinical integration (should the trials be successful) would be beneficial. These elements should form part of the full application.

Feedback to applicants

- Please provide constructive comments/suggestions suitable to be fed back to the applicant to help form a full application. These should highlight specific weaknesses and positive aspects of the application.

The applicants should be commended for their concise, clearly written application with clear research questions, objectives and methods.

As a significant amount of funding (£50,000) will be dedicated to the subcontracting of software development on this already implemented solution, it would be beneficial for the applicants to provide some further detail of the types of additional development that are envisioned. It would also be beneficial to show when this software development is anticipated to take place within Appendix A (Gantt Chart)

It is particularly interesting to see that Dr Meineart has interests in clinical artificial intelligence – will this be integrated into NMS Assist? I believe this could be an excellent source of novelty. Further detail on how NMS Assist differs from other existing approaches would be beneficial – what are the novel aspects?

It would be beneficial to provide more detail on planned recruitment. Will you recruit participants from a wide range of ages, with a wide range of symptoms and at various stages of their Parkinson's journey? The stratification of participants may provide interesting insight into who can most benefit from this intervention.

The application mentions that responses to remote monitoring that trigger additional service support will be measured – further details of this process would be beneficial.

This work is of clear importance, however, I do feel the application would benefit from further insight into the novelty of this work, and evidence-based potential benefits to quality of life / clinical intervention / costs that may result. Additionally, further insight into anticipated pathway to clinical integration (should the trials be successful) would be beneficial. These elements should form part of the full application.

Any other comments

Score: 7/10

Please send your completed review to researchapplications@parkinsons.org.uk

Funding category	Characteristics for Scientific Members	Rating scale	
**Highly fundable**	• Very important research questions; likely to result in advancement in the scientific understanding of Parkinson's or significant benefit for people affected by Parkinson's by addressing the priority research areas for improving everyday life.<br>• Excellent and appropriate methods and research design.<br>• Very strong, internationally competitive team, containing all relevant disciplines.<br>• Very good value for money.<br>• Clear and well written pre-proposal.<br>• Plain English summary accurately reflects the research proposal and is realistic about potential outcomes and timescales involved.	Exceptional	**10**
		Excellent quality research	**9**
		Very good, bordering on excellent	**8**
**Potentially fundable**	• Relevant research questions; likely to result in advancement in the scientific understanding of Parkinson's, or benefit for people affected by Parkinson's by addressing the priority research areas for improving everyday life.<br>• Good quality and appropriate methods and research design.<br>• Competent and appropriate research team containing all key disciplines.<br>• Good value for money.<br>• Plain English summary accurately reflects the research proposal and is realistic about potential outcomes and timescales involved.	Good quality research	**7**
		Above average quality research	**6**
		Acceptable quality	**5**
**Not fundable (without significant changes)**	• Research questions are not directly relevant to the scientific understanding of Parkinson's or do not address the priority research areas for people affected by Parkinson's.<br>• Inappropriate methods and research design of only modest or poor quality.<br>• Applicants without relevant research experience or key disciplines not represented.<br>• Poor value for money.<br>• Plain English summary is unclear, does not accurately reflect the research proposal and is unrealistic about the potential outcomes and timescales involved.	Borderline quality research	**4**
		Below acceptable quality	**3**
**Definitely not fundable**	• Irrelevant research questions.<br>• Poor/flawed/duplicative methods and research design.<br>• Key skills missing from the research team.<br>• Very poor value for money.<br>• Unclear application.<br>• Plain English summary is unclear, does not accurately reflect the research proposal and is unrealistic about the potential outcomes and timescales involved.	Many identified flaws	**2**
		Serious weaknesses or major concerns	**1**

Project Grants - Parkinson's UK College of Experts scoring system

The listed characteristics are for guidance only. The characteristics are general statements on the overall quality of the application in each funding category. They are not a checklist of minimum criteria for the funding category.

Parkinson’s UK top 10 priority research areas for improving everyday life

1. Balance and falls
2. Stress and anxiety
3. Uncontrollable movements
4. Personalised treatments
5. Dementia
6. Mild thinking and memory problems
7. Monitoring symptoms
8. Sleep
9. Dexterity
10. Urinary problems

For more information on the priority research areas and the full list, please visit our website.

College of Experts
Non drug approaches grant pre-proposal reviewer form

PARKINSON'S[UK]
CHANGE ATTITUDES.
FIND A CURE.
JOIN US.

This form should be completed by the College of Expert reviewer for a non drug approaches grant pre-proposal. The information you provide is maintained as a record of the decision-making process and to give feedback to applicants.

If you have any questions, please contact the Research team by emailing researchapplications@parkinsons.org.uk.

**Application reference (Internal use)**	NDA-21-15	**Reviewer reference (Internal use)**	
**Principal applicant**	Dr Edward Meinert<br>Dr Camille Carroll		
**Application title**	The impact of a digital portal on the monitoring and self-management of nonmotor symptoms in people with Parkinson's		

Please do remember to provide a score for your evaluation at the end of this review.

Importance

- How important are the questions, or gaps in knowledge, that are being addressed? Is there a good rationale for pursuing these? Is success likely to lead to significant new understanding?
- Is the research relevant to Parkinson's?
- Does the proposal realistically set out the ultimate potential health or social gain to people with Parkinson's?
- How important is it to do the work now?
- Is there similar or complementary research underway elsewhere? Are the proposals competitive?

The monitoring and assessment of non-motor symptoms is notoriously difficult to assess and has considerable impact on People with Parkinson's (PwP) quality of life. The proposed study aims to assess the benefits of a digital tool that has been developed to support the monitoring and self-management of non-motor symptoms, as well as providing a means of accessing resources to aid management of these. As well as potentially improving the quality of life of PwP, a health economic assessment is proposed to understand better the potential cost benefits of such a system. A fuller consideration of any competing technologies would have been useful.

Environment and people

- Have the applicants established a high-quality track record in the field, including publication history?
- Are the applicants uniquely placed to deliver the work?
- Where the proposal embarks on work in a field new to the applicants, or is a first funding application, is there a firm foundation to take the work forward?
- How well does the work fit with other relevant research pursued by the applicants?

Parkinson's UK is the operating name of the Parkinson's Disease Society of the United Kingdom. A company limited by guarantee. Registered in England and Wales (948776). Registered office: 215 Vauxhall Bridge Road, London SW1V 1EJ. A charity registered in England and Wales (258197) and in Scotland (SC037554).

Both of the applicants have highly relevant and extensive research experience in their respective fields, as evidenced by many peer reviewed publications to date and are therefore well placed to deliver the work proposed. They are supported by experienced technologists and health professionals.

Research plans

- Will the proposed plan of work achieve the objectives outlined in the proposal?
- Are the methodological plans realistic, given the aims of the research and the resources?
- Are the methods and study designs appropriate and free of flaws?

The aims and objectives of the proposed research are clearly stated and justified. These are detailed in the respective work packages and summary work plan; they are well justified and scheduled in the context of a realistic timescale. A clear methodology has been adopted to ensure rigorous investigation can be achieved.

Justification of resources requested

- Is the number of staff appropriate for the work described? Are the reasons for purchasing major items of equipment clearly set out?
- Are the funds requested appropriate for the work proposed and does the scientific potential justify funding on the scale requested? Is the project value for money?
- Are the timescale and scheduling realistic?

A good balance between research staff, administrative support and consultancy has been proposed to achieve the most effective use of resources. I believe that the work proposed does justify funding as requested and, importantly, is realistic in what is required to achieve a successful outcome in the timescale proposed. It is, therefore, good value for money.

Evidence of potential impact and clear pathway for benefit to people with Parkinson’s in the near future

With these awards we are focused on delivering outcomes that make a difference to people with the condition. That means that we're looking for projects that, if successful, have a clear pathway to be made available to people affected by Parkinson's.

Please let us know whether this is apparent in this pre proposal application

If the outcome of this project is that the digital tool is effective in supporting the monitoring and self-management of non-motor symptoms, it will be of considerable benefit to PwP in the short to medium term.

Feedback to applicants

- Please provide constructive comments/suggestions suitable to be fed back to the applicant to help form a full application. These should highlight specific weaknesses and positive aspects of the application.

A well justified and compelling proposal that gives confidence a thorough investigation into the benefits of the digital technology will be rigorously investigated.

A fuller consideration of any competing technologies would have been useful.

Any other comments

My assessment is: Excellent quality research 9

.

Please send your completed review to researchapplications@parkinsons.org.uk

Funding category	Characteristics for Scientific Members	Rating scale	
**Highly fundable**	• Very important research questions; likely to result in advancement in the scientific understanding of Parkinson's or significant benefit for people affected by Parkinson's by addressing the priority research areas for improving everyday life.<br>• Excellent and appropriate methods and research design.<br>• Very strong, internationally competitive team, containing all relevant disciplines.<br>• Very good value for money.<br>• Clear and well written pre-proposal.<br>• Plain English summary accurately reflects the research proposal and is realistic about potential outcomes and timescales involved.	Exceptional	**10**
		Excellent quality research	**9**
		Very good, bordering on excellent	**8**

**Potentially fundable**	• Relevant research questions; likely to result in advancement in the scientific understanding of Parkinson's, or benefit for people affected by Parkinson's by addressing the priority research areas for improving everyday life.<br>• Good quality and appropriate methods and research design.<br>• Competent and appropriate research team containing all key disciplines.<br>• Good value for money.<br>• Plain English summary accurately reflects the research proposal and is realistic about potential outcomes and timescales involved.	Good quality research	**7**
		Above average quality research	**6**
		Acceptable quality	**5**
**Not fundable (without significant changes)**	• Research questions are not directly relevant to the scientific understanding of Parkinson's or do not address the priority research areas for people affected by Parkinson's.<br>• Inappropriate methods and research design of only modest or poor quality.<br>• Applicants without relevant research experience or key disciplines not represented.<br>• Poor value for money.<br>• Plain English summary is unclear, does not accurately reflect the research proposal and is unrealistic about the potential outcomes and timescales involved.	Borderline quality research	**4**
		Below acceptable quality	**3**
**Definitely not fundable**	• Irrelevant research questions.<br>• Poor/flawed/duplicative methods and research design.<br>• Key skills missing from the research team.<br>• Very poor value for money.<br>• Unclear application.<br>• Plain English summary is unclear, does not accurately reflect the research proposal and is unrealistic about the potential outcomes and timescales involved.	Many identified flaws	**2**
		Serious weaknesses or major concerns	**1**

Project Grants - Parkinson's UK College of Experts scoring system

The listed characteristics are for guidance only. The characteristics are general statements on the overall quality of the application in each funding category. They are not a checklist of minimum criteria for the funding category.

Parkinson's UK top 10 priority research areas for improving everyday life

1 Balance and falls

2 Stress and anxiety

3 Uncontrollable movements

4 Personalised treatments

5 Dementia

6 Mild thinking and memory problems

7 Monitoring symptoms

8. Sleep
9. Dexterity
10. Urinary problems

For more information on the priority research areas and the full list, please visit our website.

Non drug approaches pre-proposal review form for lay grant reviewers

PARKINSON'S UK
CHANGE ATTITUDES.
FIND A CURE.
JOIN US.

Thank you for agreeing to review a Parkinson's UK research grant application.

With these awards we are focused on delivering outcomes that make a difference to people with Parkinson's. That means that we're looking for projects that, if successful, have a clear pathway to be made available to people affected by Parkinson's. Find out more about the scheme

**Application reference**	NDA 21-15
**Principal applicant**	Dr. Edward Meinert Dr Camille Carroll
**Application title**	The impact of a digital portal on the monitoring and self management of non-motor symptoms in people with Parkinson's.

1. Do you think that the proposed project is important to people affected by Parkinson's?

Yes

We want to know if the project is important to people affected by Parkinson's. And whether it is likely to deliver impact or improvement to the lives of people affected by Parkinson's.

Has the potential to make assessments more timely, thorough, relevant and cost effective in a non intrusive way.

2. Does the proposed project include a clear pathway to being made available to people affected by Parkinson's?

Yes

Does involve Plymouth Parkinson's service. Mention of PPI but unclear about recruitment, ease of access and time commitment etc.

3. Do you think the applicants should be invited to submit a full application?

Yes

Full application will need to address recruitment issues, time commitment and how PPI involvement will be taken into account.

Score

The listed characteristics are for guidance only. The characteristics are general statements on the overall quality of the application in each in funding category. They are not a checklist of minimum criteria for the funding category. **Please tick the box next to the score which best reflects your views.**

Funding category	Characteristics	Rating scale		
**Highly fundable**	• Clear and well written proposal.<br>• Very important research questions; likely to result in significant benefit for people affected by Parkinson's.<br>• Strong evidence of meaningful and well-planned patient and public involvement, with activities integrated at relevant points throughout the project.	Exceptional	10	
		Excellent quality research	9	
		Very good, bordering on excellent	8	
**Potentiall y fundable**	• Relevant research questions; likely to result in benefit for people affected by Parkinson's.<br>• All key aspects of application are clearly presented.<br>• Some evidence of patient and public involvement, with activities well planned and integrated at relevant points.	Good quality research	7	
		Above average quality research	6	X
		Acceptable quality	5	
**Not fundable (without significan t changes)**	• Research questions are not directly relevant to Parkinson's or are unlikely to result in benefit for people affected by Parkinson's.<br>• Key elements of the application are unclear.<br>• Limited evidence of patient and public involvement with unclear plans.	Borderline quality research	4	
		Below acceptable quality	3	
**Definitely not fundable**	• Irrelevant research questions.<br>• Unclear application.<br>• No or limited evidence of appropriate patient and public involvement in the research.	Many identified flaws	2	
		Serious weaknesse s or major concerns	1	

Parkinson's UK top 10 priority areas for improving everyday life

For more information on the priority research areas and the full list, please visit our website.

**1**	**Balance and falls**	**6**	**Mild thinking and memory problems**
**2**	**Stress and anxiety**	**7**	**Monitoring symptoms**
**3**	**Uncontrollable movements**	**8**	**Sleep**
**4**	**Personalised treatments**	**9**	**Dexterity**
**5**	**Dementia**	**10**	**Urinary problems**

Please tick here if you would be happy for us to use anonymised versions of your comments for future training and feedback purposes.	X

Please email the completed review forms to: researchapplications@parkinsons.org.uk

Once we have shortlisted the pre-proposal applications based on lay reviews, we will let you know which applications have been invited to submit a full application. Pre-proposal applications which are not reviewed favourably by the lay grant reviewers, will not be invited to submit a full application.

When we have received the full applications, we will invite you to review the same applications as you reviewed in the pre-proposal stage.

If you have any other questions, we are always happy to help.

PARKINSON'S[UK]
CHANGE ATTITUDES.
FIND A CURE.
JOIN US.

College of Experts
Non drug approaches grant pre-proposal reviewer form

This form should be completed by the College of Expert reviewer for a non drug approaches grant pre-proposal. The information you provide is maintained as a record of the decision-making process and to give feedback to applicants.

If you have any questions, please contact the Research team by emailing researchapplications@parkinsons.org.uk.

Application reference (Internal use)	NDA-21-15	Reviewer reference (Internal use)	
Principal applicant	Dr Edward Mienert		
Application title	The impact of a digital portal on the monitoring and self-management of nonmotor symptoms in people with Parkinson's		

Please do remember to provide a score for your evaluation at the end of this review.

Importance

- How important are the questions, or gaps in knowledge, that are being addressed? Is there a good rationale for pursuing these? Is success likely to lead to significant new understanding?
- Is the research relevant to Parkinson's?
- Does the proposal realistically set out the ultimate potential health or social gain to people with Parkinson's?
- How important is it to do the work now?
- Is there similar or complementary research underway elsewhere? Are the proposals competitive?

While the design of this study fits within the standard framework for usability, it studies only a single platform: the NMS Assist web system. This is one among a very large number of potential web-based data entry systems which could be programmed for the task of collecting data on non-motor symptoms. Because technology moves fast, any single system like this programmed today, will likely be obsolete in a year or even less. Therefore, the main criticism I would raise is that from this research, not much can be learned in about the enduring principles, if any, for creating systems which use digital data capture to improve the lives of those with Parkinson's. This is an observational study and does not address a particular digital intervention, on a small, selective cohort. These shortcomings mean that the research does not seem like a cost-effective way of leading to improved outcomes for people with Parkinson's.

Environment and people

- Have the applicants established a high-quality track record in the field, including publication history?
- Are the applicants uniquely placed to deliver the work?
- Where the proposal embarks on work in a field new to the applicants, or is a first funding application, is there a firm foundation to take the work forward?
- How well does the work fit with other relevant research pursued by the applicants?

The team have extensive experience in running observational technology studies such as this and the environment at Plymouth appears to be well set up to support the research.

Research plans

- Will the proposed plan of work achieve the objectives outlined in the proposal?
- Are the methodological plans realistic, given the aims of the research and the resources?
- Are the methods and study designs appropriate and free of flaws?

As a small cohort study the design appears sound. However, there are no sample size computations included and therefore it is hard to decide whether the quantitative part of the study would really be effectively powered. More problematic however, is that the study is purely observational, so it is unlikely to be able to address the stated question of whether the app really is leading to improved self-management, and/or prompting adjustment in care.

Justification of resources requested

- Is the number of staff appropriate for the work described? Are the reasons for purchasing major items of equipment clearly set out?
- Are the funds requested appropriate for the work proposed and does the scientific potential justify funding on the scale requested? Is the project value for money?
- Are the timescale and scheduling realistic?

Given the scale of the proposal, the costs and timescales associated with organizing quite a large team for this project are certainly to be expected. However, the substantial cost for the limited reward does seem excessive.

Evidence of potential impact and clear pathway for benefit to people with Parkinson’s in the near future

With these awards we are focused on delivering outcomes that make a difference to people with the condition. That means that we're looking for projects that, if

successful, have a clear pathway to be made available to people affected by Parkinson's.

Please let us know whether this is apparent in this pre proposal application

As this is designed as a feasibility study, it is quite early in the research for firm conclusions to be drawn about the applicability of the new NMS Assist platform. This is to be expected, but if the research does not show a positive benefit from this platform, then the research would largely have been wasted as it is so highly specific.

Feedback to applicants

- Please provide constructive comments/suggestions suitable to be fed back to the applicant to help form a full application. These should highlight specific weaknesses and positive aspects of the application.

Any other comments

Please send your completed review to researchapplications@parkinsons.org.uk

Funding category	Characteristics for Scientific Members	Rating scale	
**Highly fundable**	• Very important research questions; likely to result in advancement in the scientific understanding of Parkinson's or significant benefit for people affected by Parkinson's by addressing the priority research areas for improving everyday life.<br>• Excellent and appropriate methods and research design.<br>• Very strong, internationally competitive team, containing all relevant disciplines.<br>• Very good value for money.<br>• Clear and well written pre-proposal.<br>• Plain English summary accurately reflects the research proposal and is realistic about potential outcomes and timescales involved.	Exceptional	
		Excellent quality research	
		Very good, bordering on excellent	

**Potentially fundable**	• Relevant research questions; likely to result in advancement in the scientific understanding of Parkinson's, or benefit for people affected by Parkinson's by addressing the priority research areas for improving everyday life.<br>• Good quality and appropriate methods and research design.<br>• Competent and appropriate research team containing all key disciplines.<br>• Good value for money.<br>• Plain English summary accurately reflects the research proposal and is realistic about potential outcomes and timescales involved.	<u>Good quality research</u>	
		<u>Above average quality research</u>	
		<u>Acceptable quality</u>	
**Not fundable (without significant changes)**	• Research questions are not directly relevant to the scientific understanding of Parkinson's or do not address the priority research areas for people affected by Parkinson's.<br>• Inappropriate methods and research design of only modest or poor quality.<br>• Applicants without relevant research experience or key disciplines not represented.<br>• Poor value for money.<br>• Plain English summary is unclear, does not accurately reflect the research proposal and is unrealistic about the potential outcomes and timescales involved.	<u>Borderline quality research</u>	<u>**4**</u>
		<u>Below acceptable quality</u>	
**Definitely not fundable**	• Irrelevant research questions.<br>• Poor/flawed/duplicative methods and research design.<br>• Key skills missing from the research team.<br>• Very poor value for money.<br>• Unclear application.<br>• Plain English summary is unclear, does not accurately reflect the research proposal and is unrealistic about the potential outcomes and timescales involved.	<u>Many identified flaws</u>	
		<u>Serious weaknesses or major concerns</u>	

Project Grants - Parkinson's UK College of Experts scoring system

The listed characteristics are for guidance only. The characteristics are general statements on the overall quality of the application in each funding category. They are not a checklist of minimum criteria for the funding category.

<u>Parkinson's UK top 10 priority research areas for improving everyday life</u>

1 Balance and falls

2 Stress and anxiety

3. Uncontrollable movements
4. Personalised treatments
5. Dementia
6. Mild thinking and memory problems
7. Monitoring symptoms
8. Sleep
9. Dexterity
10. Urinary problems

For more information on the priority research areas and the full list, please visit our website.

Thank you for agreeing to review a Parkinson's UK project grant pre-proposal application.

Below each question, we have provided some key prompts for you to consider when commenting on each aspect of the application. Please feel free to make any additional comments which you think are relevant.

Your comments help us and the members of the College of Experts to understand exactly what you think about an application; and why you may or may not want to support it or think it's important to people affected by Parkinson's. It is therefore very important that you elaborate wherever possible. Your anonymised review form will be sent to applicants for their information.

General comments that could be considered vague and unspecific do not give the scientific reviewers a clear idea of what you think of the proposed research.

For more guidance please refer to the lay grant reviewer training pack and briefing document. If you have any other questions, we are always happy to help. Please call us on 020 7963 3950 or email researchapplications@parkinsons.org.uk.

**Application reference**	NDA-21-15
**Principal applicant**	Camille Carroll
**Application title**	The impact of a digital portal on the monitoring and self-management of non- motor symptoms in people with Parkinson's
**Plain English title**	
**Lay reviewer reference**	

1. What did you think of the plain English summary?

Excellent	Good ✠	Acceptable ○	Poor ○

It is really useful if lay grant reviewer's comment on the language used by researchers to explain their work in the plain English summary, as this feedback will help ensure that their future applications are accessible to those without a scientific background.

- Was the purpose of the proposed research clear?
- Did the summary help you carry out your review? If not, why not?
- Was the background to the research summarized adequately?
- Is the language used clear and understandable? Are the scientific terms and jargon well explained? If not, which terms need explanation?

Clear, easy to understand and relatively jargon free.

2. How important is this research to you as a person affected by Parkinson’s?

Please consider how important you think this area of research is to you both personally and to the wider Parkinson’s community, including carers.

- Do you think the proposed research would benefit people affected by Parkinson’s, and if yes, is this potential benefit well communicated to you in the application?
- How relevant do you think this area of research is to the priorities and needs of people affected by Parkinson’s?

We are moving into a phase of computer aided clinical management, and I think it would be beneficial for our care to investigate the effectiveness of an assessment tool such as NMS Assist. The non-motor symptoms of PD is an important field.

The entire care team, including carers are included which is very important.

5. Do you have any additional comments about the application?

If you have any additional thoughts about the ways in which the application could be improved please add them below.

Outcomes of the NMS assessment on service support and resources on patient activation management are clear and obtainable. Well thought out study.

Please tick here if you would be happy for us to use anonymised versions of your comments for future training and feedback purposes.	

Score

The listed characteristics are for guidance only. The characteristics are general statements on the overall quality of the application in each in funding category. They are not a checklist of minimum criteria for the funding category. **Please tick the box next to the score which best reflects your views.**

Funding category	Characteristics	Rating scale		
**Highly fundable**	• Clear and well written proposal.<br>• Very important research questions; likely to result in significant benefit for people affected by Parkinson's.	Exceptional	10	
		Excellent quality research	9	
		Very good, bordering on excellent	8	
**Potentially fundable**	• Relevant research questions; likely to result in benefit for people affected by Parkinson's.<br>• All key aspects of application are clearly presented.	Good quality research	7	X
		Above average quality research	6	
		Acceptable quality	5	
**Not fundable (without significant changes)**	• Research questions are not directly relevant to Parkinson's or are unlikely to result in benefit for people affected by Parkinson's.<br>• Key elements of the application are unclear.	Borderline quality research	4	
		Below acceptable quality	3	

**Definitely not fundable**	● Irrelevant research questions.<br>● Unclear application.	Many identified flaws	2	
		Serious weaknesses or major concerns	1	

Parkinson's UK top 10 priority areas for improving everyday life

For more information on the priority research areas and the full list, please visit our website.

**1**	**Balance and falls**	**6**	**Mild thinking and memory problems**
**2**	**Stress and anxiety**	**7**	**Monitoring symptoms**
**3**	**Uncontrollable movements**	**8**	**Sleep**
**4**	**Personalised treatments**	**9**	**Dexterity**
**5**	**Dementia**	**10**	**Urinary problems**

Please email the completed review forms to: researchapplications@parkinsons.org.uk.

.

Non drug approaches pre-proposal review form for lay grant reviewers

PARKINSON'S UK
CHANGE ATTITUDES.
FIND A CURE.
JOIN US.

Thank you for agreeing to review a Parkinson's UK research grant application.

With these awards we are focused on delivering outcomes that make a difference to people with Parkinson's. That means that we're looking for projects that, if successful, have a clear pathway to be made available to people affected by Parkinson's. Find out more about the scheme

**Application reference**	NDA-21-15
**Principal applicant**	Dr Edward Meinert<br>Dr Camille Carroll
**Application title**	The impact of a digital portal on the monitoring and self-management of nonmotor symptoms in people with Parkinson's

**1. Do you think that the proposed project is important to people affected by Parkinson's?**						
**Yes**	✓	**No**				

We want to know if the project is important to people affected by Parkinson's. And whether it is likely to deliver impact or improvement to the lives of people affected by Parkinson's.

The project focuses on non-motor symptoms. The app allows PwP a certain amount of self-monitoring/control of their symptoms. It allows their clinicians to view their data and propose changes. It also allows the PwP to flag concerns to their care teams. Having this ability would be very good for the PwP but I doubt that clinicians/care team would have the time to review every patients' data or be willing to have patients contact them.

2. Does the proposed project include a clear pathway to being made available to people affected by Parkinson's?

Yes **No** ✓

There is no clear pathway to being made available as it stops at the point that if successful it goes to a larger trial which will take yet more time and yet more money.

3. Do you think the applicants should be invited to submit a full application?

Yes ✓ **No**

Yes but we need a fixed being-made-available date not just a call for yet more work. We want it now. Can it be speeded up?

Score

The listed characteristics are for guidance only. The characteristics are general statements on the overall quality of the application in each in funding category. They are not a checklist of minimum criteria for the funding category. **Please tick the box next to the score which best reflects your views.**

Funding category	Characteristics	Rating scale	
**Highly fundable**	• Clear and well written proposal.<br>• Very important research questions; likely to result in significant benefit for people affected by Parkinson's.<br>• Strong evidence of meaningful and well-planned patient and public involvement, with activities integrated at relevant points throughout the project.	Exceptional	10
		Excellent quality research	9
		Very good, bordering on excellent	8
**Potentially fundable**	• Relevant research questions; likely to result in benefit for people affected by Parkinson's.<br>• All key aspects of application are clearly presented.<br>• Some evidence of patient and public involvement, with activities well planned and integrated at relevant points.	Good quality research	7 ✔
		Above average quality research	6
		Acceptable quality	5
**Not fundable (without significant changes)**	• Research questions are not directly relevant to Parkinson's or are unlikely to result in benefit for people affected by Parkinson's.<br>• Key elements of the application are unclear.<br>• Limited evidence of patient and public involvement with unclear plans.	Borderline quality research	4
		Below acceptable quality	3
**Definitely not fundable**	• Irrelevant research questions.<br>• Unclear application.<br>• No or limited evidence of appropriate patient and public involvement in the research.	Many identified flaws	2
		Serious weaknesses or major concerns	1

Parkinson's UK top 10 priority areas for improving everyday life

For more information on the priority research areas and the full list, please visit our website.

1	**Balance and falls**	6	**Mild thinking and memory problems**
2	**Stress and anxiety**	7	**Monitoring symptoms**
3	**Uncontrollable movements**	8	**Sleep**
4	**Personalised treatments**	9	**Dexterity**
5	**Dementia**	10	**Urinary problems**

Please tick here if you would be happy for us to use anonymised versions of your comments for future training and feedback purposes.	✔

Please email the completed review forms to: researchapplications@parkinsons.org.uk

Once we have shortlisted the pre-proposal applications based on lay reviews, we will let you know which applications have been invited to submit a full application. Pre-proposal applications which are not reviewed favourably by the lay grant reviewers, will not be invited to submit a full application.

When we have received the full applications, we will invite you to review the same applications as you reviewed in the pre-proposal stage.

If you have any other questions, we are always happy to help.
