# Supplementary material for: The Impact of a Digital Artificial Intelligence System on the Monitoring and Self-management of Nonmotor Symptoms in People With Parkinson Disease: Proposal for a Phase 1 Implementation Study
Source: JMIR Res Protoc. 2022 Sep 26;11(9):e40317. doi: 10.2196/40317 (PMC9555326; doi:10.2196/40317)
Supplement: Multimedia Appendix 3 [file resprot_v11i9e40317_app3.pdf]

Peer review of a grant application

PARKINSON'S UK
CHANGE ATTITUDES.
FIND A CURE.
JOIN US.

Application reference	NDA-21-04	Reviewer reference	B
Principal applicant	Dr Edward Meinert		
Application title	The impact of a digital system on the monitoring and self-management of non-motor symptoms in People with Parkinson's		

Importance

- How important are the questions, or gaps in knowledge, that are being addressed? Is there a good rationale for pursuing these? Is success likely to lead to significant new understanding?
- Is the research relevant to Parkinson's?
- Does the proposal realistically set out the ultimate potential health or social gain to people with Parkinson's?
- How important is it to do the work now?
- Is there similar or complementary research underway elsewhere? Are the proposals competitive?

The monitoring and assessment of non-motor symptoms are notoriously difficult to assess and have a considerable impact on People with Parkinson's (PwP) quality of life, so research in this area is important and needed. The proposed study aims to assess the benefits of a digital tool that has been developed to support the monitoring and self-management of non-motor symptoms, as well as providing a means of accessing resources to aid the management of these and is highly relevant to Parkinson's. As well as potentially improving the quality of life of PwP, a health economic assessment is proposed to understand better the potential cost benefits of such a system. A detailed comparison with competing technologies has now been provided setting out the benefits of the proposed system and its potential benefits not only to PwP but also the Care-Partner (CP) and healthcare professionals. A clear case is also made of how funding by Parkinson's UK will support the timely development and evaluation of the technology towards adoption.

Environment and people

- Have the applicants established a high-quality track record in the field, including publication history?
- Are the applicants uniquely placed to deliver the work?
- Where the proposal embarks on work in a field new to the applicants, or is a first funding application, is there a firm foundation to take the work forward?
- How well does the work fit with other relevant research pursued by the applicants?

The applicants have highly relevant and extensive research experience in their respective fields, as evidenced by the many relevant peer-reviewed publications and previously funded research projects. They are supported by an extensive network of PwP, experienced technologists, health professionals, and other relevant personal. This provides a high level

Parkinson's UK is the operating name of the Parkinson's Disease Society of the United Kingdom. A company limited by guarantee. Registered in England and Wales (948776). Registered office: 215 Vauxhall Bridge Road, London SW1V 1EJ. A charity registered in England and Wales (258197) and in Scotland (SC037554).

of confidence that they are well placed to deliver the work proposed.

Research plans

- Will the proposed plan of work achieve the objectives outlined in the proposal?
- Are the methodological plans realistic, given the aims of the research and the resources?
- Are the methods and study designs appropriate and free of flaws?
- Is the sample size appropriate? (if applicable)
- Have major scientific, technical or organisational challenges been identified and solutions been given?

The aims and objectives of the proposed research are clearly stated and justified. These are detailed in the respective work packages and summary work plan; they are well justified and scheduled in the context of a realistic timescale. A clear methodology has been adopted to ensure rigorous investigation can be achieved. The sample size is appropriate for this stage of evaluation and feasible within the timeframe and resources requested. The significant complexity of undertaking a study of this type has been comprehensively addressed.

Justification of resources requested

- Is the number of staff appropriate for the work described? Are the reasons for purchasing major items of equipment clearly set out?
- Are the funds requested appropriate for the work proposed and does the scientific potential justify funding on the scale requested? Is the project value for money?
- Are the timescale and scheduling realistic?

A good balance between research staff, administrative support and consultancy has been proposed to achieve the most effective use of resources. I believe that the work proposed does justify the funding as requested and, importantly, is realistic in what is required to achieve a successful outcome in the timescale proposed. It is, therefore, good value for money.

Have the researchers demonstrated Patient and Public Involvement (PPI) in shaping their research? (if applicable)

It is recognised that the best quality Parkinsonâ€™s research is carried out when researchers work **with** patients and carers in the planning, delivery and/or dissemination of their research. This is called Patient and Public Involvement (PPI). PPI can include, for example, people affected by Parkinsonâ€™s working with researchers to identify the most relevant research questions, comment on the design of a study, develop recruitment and retention plans for participants, or offer advice as members of a project steering group.

- Do you think there are any strengths or weaknesses to how the research team have or plan to use PPI in their study? Do you have any suggestions for improvements?
- Do you think the resources and arrangements (such as travel/subsistence expenses and staff time) allocated for PPI in their project are sufficient and appropriate; and do you think people would be willing to work with the researchers as a result?
- If the researchers have not undertaken any PPI â€“ do you have any recommendations for how people affected by Parkinsonâ€™s could be involved in the study going forwards?

The approach to PPI is exemplary and an example of good practice. A clear strategy of involving PwP, CP and other stakeholders from the conception of the project through to completion has been followed and evident in the study design.

Ethical implications

- Are there any ethical issues that need separate consideration?

All necessary ethical issues have been clearly stated - no separate consideration is required.

Use of animals and 3Rs (for research involving animals)

The 3Rs principles are:
Replace the use of animals with alternative techniques, or avoid the use of animals altogether
Refine the way experiments are carried out, to make sure animals suffer as little as possible. This includes better housing and improvements to procedures which minimise pain and suffering and/or improve animal welfare.
Reduce the number of animals used to a minimum by seeking ways to find out information from fewer animals or more information from the same number of animals.

- Can the research question be addressed without the use of animals, or by using a less sentient species?
- Are the experimental design and the numbers of animals proposed to be used appropriate and justified; are sample size calculations correct?
- Is the expected severity of the procedures accurate and justified?
- Would any of the studies not be considered acceptable in your laboratory; if not, why not?
  - Could the research approach be improved to replace some use of animals, reduce the number of animals used, and/or refine procedures and husbandry to reduce animal pain, suffering or distress? (For 3Rs opportunities and advice, see the NC3Rs website).
  - Whether more modern, less-invasive methods can be used in place of traditional, invasive approaches procedures on animals
  - What method of restraint will be used? Is this appropriate?
  - Are appropriate humane endpoints being used to limit unnecessary suffering?
  - Could animals be sourced locally to avoid long, multi-staged transport?
  - Are requests for additional animals as a contingency adequately justified?
  - Do the staff members and researchers involved in the study have sufficient experience and training in animal use, care and welfare?
- Will the study lead to replacement, refinement and reduction in the use of animals in the future? If so, how will the findings be disseminated?
- Will publications arising from the research adhere to the ARRIVE guidelines? (Note that this applies to the reporting of all in vivo research, not just the 3Râ€™s implications).

N/A

Feedback to applicants

- Please provide constructive comments/suggestions suitable to be fed back to the applicant. These should highlight specific weaknesses and positive aspects of the application.

This is a convincing and well-written proposal that has real potential to improve the lives of People with Parkinson's and their Care-Partners. Many of the reviewers' comments have been adequately addressed which has resulted in a substantially enhanced application. My only suggestion for further consideration is additional ways in which further development of the technology, following a successful outcome of the proposed work, may be attained through commercialisation or private investment.

Any other comments

My assessment is: Excellent quality research 9

Declaration of interest: I was previously involved in a clinical sub-study of the PD-STAT trial which was led by Co-Investigator, Dr Carroll.

Score

The listed characteristics are for guidance only. The characteristics are general statements on the overall quality of the application in each in funding category. They are not a checklist of minimum criteria for the funding category.

Funding category	Characteristics	Rating scale		
**Highly fundable**	• Very important research questions; likely to result in advancement in the scientific understanding of Parkinsonâ€™s or significant benefit for people affected by Parkinsonâ€™s by addressing the priority research areas for improving everyday life.<br>• Excellent and appropriate methods and research design.<br>• Very strong, internationally competitive team, containing all relevant disciplines.<br>• Very good value for money.<br>• Clear and well written proposal.<br>• Strong evidence of meaningful and well-planned patient and public involvement, with activities integrated at appropriate points throughout the project.<br>• Plain English summary accurately reflects the research proposal and is realistic about potential outcomes and timescales involved.	Exceptional	10	
		Excellent quality research	9	X
		Very good, bordering on excellent	8	
**Potentially fundable**	• Relevant research questions; likely to result in advancement in the scientific understanding of Parkinsonâ€™s, or benefit for people affected by Parkinsonâ€™s by addressing the priority research areas for improving everyday life.<br>• Good quality and appropriate methods and research design.<br>• Competent and appropriate research team containing all key disciplines.<br>• Good value for money.<br>• All key aspects of application are clearly presented<br>• Some evidence of patient and public involvement, with activities well planned and integrated at appropriate points.<br>• Plain English summary accurately reflects the research proposal and is realistic about potential outcomes and timescales involved.	Good quality research	7	
		Above average quality research	6	
		Acceptable quality	5	
**Not fundable (without significant**	• Research questions are not directly relevant to the scientific understanding of Parkinsonâ€™s or do not address the priority research areas for people affected by Parkinsonâ€™s.<br>• Inappropriate methods and research design of only modest or poor quality.<br>• Applicants without relevant research experience or key disciplines not represented.<br>• Poor value for money.<br>• Key elements of the application are unclear.	Borderline quality research	4	
		Below		

**changes)**	• Limited evidence of patient and public involvement with unclear plans.<br>• Plain English summary is unclear, does not accurately reflect the research proposal and is unrealistic about the potential outcomes and timescales involved.	acceptable quality	3	
**Definitely not fundable**	• Irrelevant research questions.<br>• Poor/flawed/duplicative methods and research design.<br>• Key skills missing from the research team.<br>• Very poor value for money.<br>• Unclear application.<br>• No or limited evidence of appropriate patient and public involvement in the research.<br>• Plain English summary is unclear, does not accurately reflect the research proposal and is unrealistic about the potential outcomes and timescales involved.	Many identified flaws	2	
		Serious weaknesses or major concerns	1	

Parkinsonâ€™s UK top 10 priority areas for improving everyday life

For more information on the priority research areas and the full list, please visit our website.

1. **Balance and falls**
2. **Stress and anxiety**
3. **Uncontrollable movements**
4. **Personalised treatments**
5. **Dementia**
6. **Mild thinking and memory problems**
7. **Monitoring symptoms**
8. **Sleep**
9. **Dexterity**
10. **Urinary problems**

Peer review of a grant application

PARKINSON'S UK
CHANGE ATTITUDES.
FIND A CURE.
JOIN US.

**Application reference**	NDA-21-04	**Reviewer reference**	C
**Principal applicant**	Dr Edward Meinert		
**Application title**	The impact of a digital system on the monitoring and self-management of non-motor symptoms in People with Parkinson's		

Importance

- How important are the questions, or gaps in knowledge, that are being addressed? Is there a good rationale for pursuing these? Is success likely to lead to significant new understanding?
- Is the research relevant to Parkinson's?
- Does the proposal realistically set out the ultimate potential health or social gain to people with Parkinson's?
- How important is it to do the work now?
- Is there similar or complementary research underway elsewhere? Are the proposals competitive?

Non-Motor Symptoms of Parkinson's disease are an important aspect with a significant impact on quality of life, yet have been said to have received less focus than motor symptoms in terms of innovative interventions and management. The applicants provide a solid rationale for pursuing this relevant area, with a genuine potential to have a long-term impact on the wellbeing and quality of life of individuals with these symptoms. The additional inclusion of monitoring and support for care partner wellbeing is a very positive element of the study, as is the plan to ensure the tool remains free to use for people with Parkinson's, and care partners. The applicants highlight existing work, and describe how their proposal provides a more holistic and feature-rich approach which has been co-created and designed with people affected by Parkinson's.

I am concerned that a significant portion of funding (£51,343) is allocated primarily to the development of voice access via natural language processing. The applicants do not justify this large expense, and do not indicate why this expensive feature is of importance, or how much benefit it is likely to be for people with Parkinson's.

Environment and people

- Have the applicants established a high-quality track record in the field, including publication history?
- Are the applicants uniquely placed to deliver the work?
- Where the proposal embarks on work in a field new to the applicants, or is a first funding application, is there a firm foundation to take the work forward?
- How well does the work fit with other relevant research pursued by the applicants?

The applicants are well placed to produce this work, and have a good track record of publications in the area. The team demonstrates a wide range of relevant backgrounds,

Parkinson's UK is the operating name of the Parkinson's Disease Society of the United Kingdom. A company limited by guarantee. Registered in England and Wales (948776). Registered office: 215 Vauxhall Bridge Road, London SW1V 1EJ. A charity registered in England and Wales (258197) and in Scotland (SC037554).

required to produce impactful work within this area. The submission demonstrates a logical next step in the project, which has already successfully completed various development, piloting, and refinement stages.

Research plans

- Will the proposed plan of work achieve the objectives outlined in the proposal?
- Are the methodological plans realistic, given the aims of the research and the resources?
- Are the methods and study designs appropriate and free of flaws?
- Is the sample size appropriate? (if applicable)
- Have major scientific, technical or organisational challenges been identified and solutions been given?

The proposal provides a clear and detailed overview of the objectives and steps to be taken. I believe the applicants will be capable of achieving these objectives, given the realistic and sound methodological approach which has been detailed.
The application states that 60 participants including PwP, CPs, HCPs will be recruited. It then says that at least 60 PwP and CPs will be included until demographic saturation of participant types is reached. This is somewhat unclear. Is there an upper number to how many will be feasible to recruit? How many HCPs will be recruited?

Justification of resources requested

- Is the number of staff appropriate for the work described? Are the reasons for purchasing major items of equipment clearly set out?
- Are the funds requested appropriate for the work proposed and does the scientific potential justify funding on the scale requested? Is the project value for money?
- Are the timescale and scheduling realistic?

The costs incurred are largely appropriate, and the number of staff employed is appropriate for the work described. However, the most significant cost is £51,343 for software development, which the applicants note will primarily focus on integration of voice access and natural language processing. Given that this equates to just under half of the requested budget, I would anticipate that reference to this functionality would be more extensive within the application. In particular, an evidence-based overview of why this feature is necessary, how it will function, and similar offerings. In particular, do these NLP-based approaches work well on individuals with PD who may require speech and language therapy? I note that the authors also state that the conversational agent within NMS Assist will form new IP from the project – again, more details are necessary to assess value for money on this component. As it stands, it just seems that this feature is a “nice to have, optional extra”, which is requiring a large portion of the funding. Further justification is required.

The timescale is appropriate, particularly as the applicants include contingency time for elements such as ethics approval. I do query if the applicants have considered potential impact of COVID on the execution of the project? Whilst it does appear that we are starting to return to normal, there is still the potential for further lockdowns or waves, which may limit the ability to meet participants in person. Perhaps some brief commentary on how this risk may be mitigated would inspire further confidence.

Have the researchers demonstrated Patient and Public Involvement (PPI) in shaping their research? (if applicable)

It is recognised that the best quality Parkinsonâ€™s research is carried out when researchers work **with** patients and carers in the planning, delivery and/or dissemination of their research. This is called Patient and Public Involvement (PPI). PPI can include, for example, people affected by Parkinsonâ€™s working with researchers to identify the most relevant research questions, comment on the design of a study, develop recruitment and retention plans for

participants, or offer advice as members of a project steering group.

- Do you think there are any strengths or weaknesses to how the research team have or plan to use PPI in their study? Do you have any suggestions for improvements?
- Do you think the resources and arrangements (such as travel/subsistence expenses and staff time) allocated for PPI in their project are sufficient and appropriate; and do you think people would be willing to work with the researchers as a result?
- If the researchers have not undertaken any PPI â€“ do you have any recommendations for how people affected by Parkinsonâ€™s could be involved in the study going forwards?

The project will include a project management team comprising people with Parkinson’s, CPs, and PPI representatives. Additionally, it is planned that people affected by Parkinson’s, and their care partners, will be included at all stages of the study – this is very positive, and will ensure the resulting solution is created with end users in mind.

Ethical implications

- Are there any ethical issues that need separate consideration?

I believe the authors have addressed any ethical implications through appropriate data handling and through a planned ethics application.

Use of animals and 3Rs (for research involving animals)

The 3Rs principles are:
Replace the use of animals with alternative techniques, or avoid the use of animals altogether
Refine the way experiments are carried out, to make sure animals suffer as little as possible. This includes better housing and improvements to procedures which minimise pain and suffering and/or improve animal welfare.
Reduce the number of animals used to a minimum by seeking ways to find out information from fewer animals or more information from the same number of animals.

- Can the research question be addressed without the use of animals, or by using a less sentient species?
- Are the experimental design and the numbers of animals proposed to be used appropriate and justified; are sample size calculations correct?
- Is the expected severity of the procedures accurate and justified?
- Would any of the studies not be considered acceptable in your laboratory; if not, why not?
  - Could the research approach be improved to replace some use of animals, reduce the number of animals used, and/or refine procedures and husbandry to reduce animal pain, suffering or distress? (For 3Rs opportunities and advice, see the NC3Rs website).
  - Whether more modern, less-invasive methods can be used in place of traditional, invasive approaches procedures on animals
  - What method of restraint will be used? Is this appropriate?
  - Are appropriate humane endpoints being used to limit unnecessary suffering?
  - Could animals be sourced locally to avoid long, multi-staged transport?
  - Are requests for additional animals as a contingency adequately justified?
  - Do the staff members and researchers involved in the study have sufficient experience and training in animal use, care and welfare?
- Will the study lead to replacement, refinement and reduction in the use of animals in the future? If so, how will the findings be disseminated?
- Will publications arising from the research adhere to the ARRIVE guidelines? (Note that this applies to the reporting of all in vivo research, not just the 3Râ€™s implications).

n/a

Feedback to applicants

- Please provide constructive comments/suggestions suitable to be fed back to the applicant. These should highlight specific weaknesses and positive aspects of the application.

Some other comments:

- The self-help elements are derived from an existing guide created by McGill University Health Centre – has permission been sought to use these materials? I note there is a copyright mark on the materials. Ideally this should have been referenced in the Intellectual Property section. As this forms a significant part of the content provided by the software, should the original creators of this material be involved in the study to ensure that the information has been distilled accurately? The content is of Canadian origin – does the content align with recommendations that are currently provided to UK audiences? This is a key question any advice which conflicts with that given by UK professionals may lead to reduced uptake / satisfaction.
- Feedback from HCP on the 'triggered healthcare contact' function will be very useful. Whilst this may of course be very beneficial in certain circumstances, does this ad-hoc communication channel work well with existing clinical protocols? Is it something that already overworked staff would be willing to engage with? Hopefully the study can answer these questions.
- Clinical decision support is mentioned as a feature, but this isn't elaborated upon within the proposal – obviously the system will provide formal questionnaire scores, but will it also provide recommendations to support decision making?
- It is mentioned that a smaller group will discuss their experience with the solution in more detail – what size will this group be, and how will those individuals be chosen? Will there be time during the project to implement any suggested changes, or will this require further funding?
- The applicants state that the project will result in a large, longitudinal research database. Such a database would provide further value for money for this funding, and would be very useful to the research community. Are there plans to make it publicly available?
- Success criteria may need to be further defined, e.g. "regular use", "low dropout rates" – perhaps this would benefit from set threshold numbers.
- This study will be followed by an efficacy-oriented trial and effectiveness-oriented trial. Have the authors given thought to the budget and funding sources for these trials?

This is an above average quality submission with a potentially impactful result. It is, however, unfortunate that further funding will be required for two further trials after this study is complete. My largest concern is over the significant amount of money allocated to the development of the natural language processing component. I feel this would need to be addressed in significantly more detail before I could confidently state that the project is value for money

Any other comments

n/a

Score

The listed characteristics are for guidance only. The characteristics are general statements on the overall quality of the application in each in funding category. They are not a checklist of minimum criteria for the funding category.

Funding category	Characteristics	Rating scale		
	• Very important research questions; likely to result in advancement in the scientific understanding of Parkinsonâ€™s or significant	Exceptional	10	
		Excellent		

**Highly fundable**	benefit for people affected by Parkinsonâ€™s by addressing the priority research areas for improving everyday life.<br>• Excellent and appropriate methods and research design.<br>• Very strong, internationally competitive team, containing all relevant disciplines.<br>• Very good value for money.<br>• Clear and well written proposal.<br>• Strong evidence of meaningful and well-planned patient and public involvement, with activities integrated at appropriate points throughout the project.<br>• Plain English summary accurately reflects the research proposal and is realistic about potential outcomes and timescales involved.	quality research	9	
		Very good, bordering on excellent	8	
**Potentially fundable**	• Relevant research questions; likely to result in advancement in the scientific understanding of Parkinsonâ€™s, or benefit for people affected by Parkinsonâ€™s by addressing the priority research areas for improving everyday life.<br>• Good quality and appropriate methods and research design.<br>• Competent and appropriate research team containing all key disciplines.<br>• Good value for money.<br>• All key aspects of application are clearly presented<br>• Some evidence of patient and public involvement, with activities well planned and integrated at appropriate points.<br>• Plain English summary accurately reflects the research proposal and is realistic about potential outcomes and timescales involved.	Good quality research	7	
		Above average quality research	6	X
		Acceptable quality	5	
**Not fundable (without significant changes)**	• Research questions are not directly relevant to the scientific understanding of Parkinsonâ€™s or do not address the priority research areas for people affected by Parkinsonâ€™s.<br>• Inappropriate methods and research design of only modest or poor quality.<br>• Applicants without relevant research experience or key disciplines not represented.<br>• Poor value for money.<br>• Key elements of the application are unclear.<br>• Limited evidence of patient and public involvement with unclear plans.<br>• Plain English summary is unclear, does not accurately reflect the research proposal and is unrealistic about the potential outcomes and timescales involved.	Borderline quality research	4	
		Below acceptable quality	3	

**Definitely not fundable**	• Irrelevant research questions.<br>• Poor/flawed/duplicative methods and research design.<br>• Key skills missing from the research team.<br>• Very poor value for money.<br>• Unclear application.<br>• No or limited evidence of appropriate patient and public involvement in the research.<br>• Plain English summary is unclear, does not accurately reflect the research proposal and is unrealistic about the potential outcomes and timescales involved.	Many identified flaws	2	
		Serious weaknesses or major concerns	1	

Parkinsonâ€™s UK top 10 priority areas for improving everyday life

For more information on the priority research areas and the full list, please visit our website.

1. **Balance and falls**
2. **Stress and anxiety**
3. **Uncontrollable movements**
4. **Personalised treatments**
5. **Dementia**
6. **Mild thinking and memory problems**
7. **Monitoring symptoms**
8. **Sleep**
9. **Dexterity**
10. **Urinary problems**

NDA-21-04: Suggested score 5/10 (Acceptable quality)

In general I think this is a good idea. The design and implementation of the digital questionnaire system addresses a clear unmet need in Parkinson's. It is a well-designed proposal which has a good chance of achieving its objectives. The technology is reliable and builds on existing infrastructure and therefore, the proposal is likely to produce results which could be useful in other contexts.

My main concern is that the experimental design, based on qualitative interviews, is somewhat of a missed opportunity. Specifically, as stated at the end of the abstract, if it is "thought that the system will result in improved QoL" then why not actually test this in a simple RCT with QoL as an outcome? This would seem to be much better value for money because at least we would get quantitative evidence on which to build further refinements of the system, or systems like it. I do understand the role and place of this kind of qualitative research, but I think, for the sake of targeted progress in this discipline, we should be more confident about moving to generate the best quality evidence that we can.

Lay review of a
research grant application

PARKINSON'S UK
CHANGE ATTITUDES.
FIND A CURE.
JOIN US.

Thank you for agreeing to review a Parkinson's UK research grant application.

Below each question, we have provided some key prompts for you to consider when commenting on each aspect of the application. Please feel free to make any additional comments which you think are relevant.

Your comments help us and the Grant Assessment Panel to understand exactly what you think about an application; and why you may or may not want to support it or think it's important to people affected by Parkinson's. It is therefore very important that you elaborate wherever possible. Your anonymised review form will be sent to applicants for their information.

General comments that could be considered vague and unspecific do not give the Grant Assessment Panel a clear idea of what you think of the proposed research.

For more guidance please refer to the lay grant reviewer training pack and briefing document. If you have any other questions, we are always happy to help. Please call us on 020 7963 9376 or email researchapplications@parkinsons.org.uk.

**Application reference**	NDA-21-04
**Principal applicant**	Dr Edward Meinert
**Application title**	The impact of a digital system on the monitoring and self-management of non-motor symptoms in People with Parkinson™s
**Plain English title**	Evaluating a digital system for supporting People with Parkinson™s disease to monitor and manage non-motor symptoms
**Lay reviewer reference**	A

1. What did you think of the plain English summary?

Excellent ○ Good ◉ Acceptable ○ Poor ○

It is really useful if lay grant reviewers comment on the language used by researchers to explain their work in the plain English summary, as this feedback will help ensure that their future applications are accessible to those without a scientific background.

- Was the purpose of the proposed research clear?
- Did the summary help you carry out your review? If not, why not?
- Was the background to the research summarized adequately?
- Is the language used clear and understandable? Are the scientific terms and jargon well explained? If not, which terms need explanation?

Clear. Adequate detail.

s2. How important is this research to you as a person affected by Parkinson's?

Please consider how important you think this area of research is to you both personally and to the wider Parkinson's community, including carers.

- Do you think the proposed research would benefit people affected by Parkinson's, and if yes, is this potential benefit well communicated to you in the application?
- How relevant do you think this area of research is to the priorities and needs of people affected by Parkinson's?

I am sure that many PwP fail to realise that their NMS are actually related to the disease, and may under report these during medical interviews. NMS assist utilises existing proven questionaires, but is novel in its concentratation on NMS. If this can lead to successful self management and greater awareness of the problem this will be of value to the PD community.

3. How do the researchers plan to recruit and retain people participating in their research? (if applicable)

Many researchers do not have personal experience of Parkinson's, so may not consider some of the practical issues that people affected by the condition face every day. It is therefore very important that any potential barriers to the participation, recruitment and retention to the study of people affected by Parkinson's are highlighted to the applicants.

- Do you think the proposed use of participants in this research is well planned?
- Do you think people would be willing to take part? Why?
- What could the researchers do to make it easier for people with Parkinson's to take part?
- Are there any practical issues that the researchers have not considered with regards to people with Parkinson's?

The reseaearch is well planned and PwP who are not antagonistic to computers should be willing to take part. Natural language (no key board skills) to complete the questionaires is a great advantage.

4. Have the researchers demonstrated Patient and Public Involvement (PPI) in shaping their research? (if applicable)

It is recognised that the best quality Parkinson's research is carried out when researchers work **with** patients and carers in the planning, delivery and/or dissemination of their research. This is called Patient and Public Involvement (PPI).

PPI can include, for example, people affected by Parkinson's working with researchers to identify the most relevant research questions, comment on the design of a study, develop recruitment and retention plans for participants, or offer advice as members of a project steering group.

- Do you think there are any strengths or weaknesses to how the research team have/ or plan to use PPI in their study? Do you have any suggestions for improvements?
- Do you think the resources and arrangements (such as travel/ subsistence expenses and staff time) allocated for PPI in their project are sufficient and appropriate; and do you think people would be willing to work with the researchers as a result?
- If the researchers have not done any PPI – do you have any recommendations for how people affected by Parkinson's could be involved in the study going forwards?

PPI well integrated in the project.

5. Do you have any additional comments about the application?

If you have any additional thoughts about the ways in which the application could be improved please add them below.

This is a linear study over a year, with analytical tools used I have no knowledge of. I do wonder about the lack of a control group. Are they going to see specialist nurse pracitioners during the study period and how is this going to be allowed for? The inclusion of the carer's perspective is very important, but a different issue I would imagine relating more to mobility and dementia.

Score

The listed characteristics are for guidance only. The characteristics are general statements on the overall quality of the application in each in funding category. They are not a checklist of minimum criteria for the funding category.

Funding category	Characteristics	Rating scale		
**Highly fundable**	• Clear and well written proposal.<br>• Very important research questions; likely to result in significant benefit for people affected by Parkinson's.<br>• Strong evidence of meaningful and well-planned patient and public involvement, with activities integrated at relevant points throughout the project.	Exceptional	10	○
		Excellent quality research	9	○
		Very good, bordering on excellent	8	○
**Potentially fundable**	• Relevant research questions; likely to result in benefit for people affected by Parkinson's.<br>• All key aspects of application are clearly presented.<br>• Some evidence of patient and public involvement, with activities well planned and integrated at relevant points.	Good quality research	7	○
		Above average quality research	6	◉
		Acceptable quality	5	○
**Not fundable (without significant changes)**	• Research questions are not directly relevant to Parkinson's or are unlikely to result in benefit for people affected by Parkinson's.<br>• Key elements of the application are unclear.<br>• Limited evidence of patient and public involvement with unclear plans.	Borderline quality research	4	○
		Below acceptable quality	3	○
**Definitely not fundable**	• Irrelevant research questions.<br>• Unclear application.<br>• No or limited evidence of appropriate patient and public involvement in the research.	Many identified flaws	2	○
		Serious weaknesses or major concerns	1	○

Parkinson’s UK top 10 priority areas for improving everyday life

For more information on the priority research areas and the full list, please visit our website.

**1**	**Balance and falls**
**2**	**Stress and anxiety**
**3**	**Uncontrollable movements**
**4**	**Personalised treatments**
**5**	**Dementia**
**6**	**Mild thinking and memory problems**
**7**	**Monitoring symptoms**
**8**	**Sleep**
**9**	**Dexterity**
**10**	**Urinary problems**

Please tick here if you would be happy for us to use anonymised versions of your comments for future training and feedback purposes ☐

Completed review forms should be emailed to: researchapplications@parkinsons.org.uk

When reviewing project grant applications, forms should also be emailed to your Lay Review Co-ordinator.

Lay review of a
research grant application

Thank you for agreeing to review a Parkinson's UK research grant application.

Below each question, we have provided some key prompts for you to consider when commenting on each aspect of the application. Please feel free to make any additional comments which you think are relevant.

Your comments help us and the Grant Assessment Panel to understand exactly what you think about an application; and why you may or may not want to support it or think it's important to people affected by Parkinson's. It is therefore very important that you elaborate wherever possible. Your anonymised review form will be sent to applicants for their information.

General comments that could be considered vague and unspecific do not give the Grant Assessment Panel a clear idea of what you think of the proposed research.

For more guidance please refer to the lay grant reviewer training pack and briefing document. If you have any other questions, we are always happy to help. Please call us on 020 7963 9376 or email researchapplications@parkinsons.org.uk.

**Application reference**	NDA-21-04
**Principal applicant**	Dr Edward Meinert
**Application title**	The impact of a digital system on the monitoring and self-management of non-motor symptoms in People with Parkinson™s
**Plain English title**	Evaluating a digital system for supporting People with Parkinson™s disease to monitor and manage non-motor symptoms
**Lay reviewer reference**	B

1. What did you think of the plain English summary?

Excellent	Good	Acceptable	Poor

It is really useful if lay grant reviewers comment on the language used by researchers to explain their work in the plain English summary, as this feedback will help ensure that their future applications are accessible to those without a scientific background.

- Was the purpose of the proposed research clear?
- Did the summary help you carry out your review? If not, why not?
- Was the background to the research summarized adequately?
- Is the language used clear and understandable? Are the scientific terms and jargon well explained? If not, which terms need explanation?

s2. How important is this research to you as a person affected by Parkinson’s?

Please consider how important you think this area of research is to you both personally and to the wider Parkinson’s community, including carers.

- Do you think the proposed research would benefit people affected by Parkinson’s, and if yes, is this potential benefit well communicated to you in the application?
- How relevant do you think this area of research is to the priorities and needs of people affected by Parkinson’s?

3. How do the researchers plan to recruit and retain people participating in their research? (if applicable)

Many researchers do not have personal experience of Parkinson’s, so may not consider some of the practical issues that people affected by the condition face every day. It is therefore very important that any potential barriers to the participation, recruitment and retention to the study of people affected by Parkinson’s are highlighted to the applicants.

- Do you think the proposed use of participants in this research is well planned?
- Do you think people would be willing to take part? Why?
- What could the researchers do to make it easier for people with Parkinson’s to take part?
- Are there any practical issues that the researchers have not considered with regards to people with Parkinson’s?

4. Have the researchers demonstrated Patient and Public Involvement (PPI) in shaping their research? (if applicable)

It is recognised that the best quality Parkinson's research is carried out when researchers work **with** patients and carers in the planning, delivery and/or dissemination of their research. This is called Patient and Public Involvement (PPI).

PPI can include, for example, people affected by Parkinson's working with researchers to identify the most relevant research questions, comment on the design of a study, develop recruitment and retention plans for participants, or offer advice as members of a project steering group.

- Do you think there are any strengths or weaknesses to how the research team have/ or plan to use PPI in their study? Do you have any suggestions for improvements?
- Do you think the resources and arrangements (such as travel/ subsistence expenses and staff time) allocated for PPI in their project are sufficient and appropriate; and do you think people would be willing to work with the researchers as a result?
- If the researchers have not done any PPI – do you have any recommendations for how people affected by Parkinson's could be involved in the study going forwards?

5. Do you have any additional comments about the application?

If you have any additional thoughts about the ways in which the application could be improved please add them below.

Score

The listed characteristics are for guidance only. The characteristics are general statements on the overall quality of the application in each in funding category. They are not a checklist of minimum criteria for the funding category.

Funding category	Characteristics	Rating scale		
**Highly fundable**	• Clear and well written proposal.<br>• Very important research questions; likely to result in significant benefit for people affected by Parkinson's.<br>• Strong evidence of meaningful and well-planned patient and public involvement, with activities integrated at relevant points throughout the project.	Exceptional	10	
		Excellent quality research	9	
		Very good, bordering on excellent	8	
**Potentially fundable**	• Relevant research questions; likely to result in benefit for people affected by Parkinson's.<br>• All key aspects of application are clearly presented.<br>• Some evidence of patient and public involvement, with activities well planned and integrated at relevant points.	Good quality research	7	
		Above average quality research	6	
		Acceptable quality	5	
**Not fundable (without significant changes)**	• Research questions are not directly relevant to Parkinson's or are unlikely to result in benefit for people affected by Parkinson's.<br>• Key elements of the application are unclear.<br>• Limited evidence of patient and public involvement with unclear plans.	Borderline quality research	4	
		Below acceptable quality	3	
**Definitely not fundable**	• Irrelevant research questions.<br>• Unclear application.<br>• No or limited evidence of appropriate patient and public involvement in the research.	Many identified flaws	2	
		Serious weaknesses or major concerns	1	

Parkinson's UK top 10 priority areas for improving everyday life

For more information on the priority research areas and the full list, please visit our website.

1	**Balance and falls**
2	**Stress and anxiety**
3	**Uncontrollable movements**
4	**Personalised treatments**
5	**Dementia**
6	**Mild thinking and memory problems**
7	**Monitoring symptoms**
8	**Sleep**
9	**Dexterity**
10	**Urinary problems**

Please tick here if you would be happy for us to use anonymised versions of your comments for future training and feedback purposes ☒

Completed review forms should be emailed to: researchapplications@parkinsons.org.uk

When reviewing project grant applications, forms should also be emailed to your Lay Review Co-ordinator.

Lay review of a
research grant application

Thank you for agreeing to review a Parkinson's UK research grant application.

Below each question, we have provided some key prompts for you to consider when commenting on each aspect of the application. Please feel free to make any additional comments which you think are relevant.

Your comments help us and the Grant Assessment Panel to understand exactly what you think about an application; and why you may or may not want to support it or think it's important to people affected by Parkinson's. It is therefore very important that you elaborate wherever possible. Your anonymised review form will be sent to applicants for their information.

General comments that could be considered vague and unspecific do not give the Grant Assessment Panel a clear idea of what you think of the proposed research.

For more guidance please refer to the lay grant reviewer training pack and briefing document. If you have any other questions, we are always happy to help. Please call us on 020 7963 9376 or email researchapplications@parkinsons.org.uk.

**Application reference**	NDA-21-04
**Principal applicant**	Dr Edward Meinert
**Application title**	The impact of a digital system on the monitoring and self-management of non-motor symptoms in People with Parkinson™s
**Plain English title**	Evaluating a digital system for supporting People with Parkinson™s disease to monitor and manage non-motor symptoms
**Lay reviewer reference**	C

1. What did you think of the plain English summary?

Excellent ☐	Good ☐	Acceptable ☐	Poor ☐

It is really useful if lay grant reviewers comment on the language used by researchers to explain their work in the plain English summary, as this feedback will help ensure that their future applications are accessible to those without a scientific background.

- Was the purpose of the proposed research clear?
- Did the summary help you carry out your review? If not, why not?
- Was the background to the research summarized adequately?
- Is the language used clear and understandable? Are the scientific terms and jargon well explained? If not, which terms need explanation?

The purpose of the research is clear. The summary did help with my review but required reading of the whole application for a fuller understanding. The figures in the research proposal section were quite informative. The background was summarized adequately. The language used was clear and understandable. Jargon and scientific terms are well explained. The list of abbreviations has 35 entries and the glossary 15 both in alphabetical order. Are Clinical services, healthcare professionals, practitioners, healthcare providers , care team and HCP the same? Do digital system,tool, app all refer to NMS Assist?

s2. How important is this research to you as a person affected by Parkinson’s?

Please consider how important you think this area of research is to you both personally and to the wider Parkinson’s community, including carers.

- Do you think the proposed research would benefit people affected by Parkinson’s, and if yes, is this potential benefit well communicated to you in the application?
- How relevant do you think this area of research is to the priorities and needs of people affected by Parkinson’s?

The proposed testing of NMS Assist would benefit PwP and also has input from carers. This comes across well particularly as there is the ability to flag up concerns to your Parkinson's specialist.
The testing of NMS Assist is of relevance to PwP and their carers giving them immediate advice and the ability to escalate concerns if necessary. However we want it now. It won't be made generally available to PwP and their carers until after successful testing over a two year period.

3. How do the researchers plan to recruit and retain people participating in their research? (if applicable)

Many researchers do not have personal experience of Parkinson’s, so may not consider some of the practical issues that people affected by the condition face every day. It is therefore very important that any potential barriers to the participation, recruitment and retention to the study of people affected by Parkinson’s are highlighted to the applicants.

- Do you think the proposed use of participants in this research is well planned?
- Do you think people would be willing to take part? Why?
- What could the researchers do to make it easier for people with Parkinson’s to take part?
- Are there any practical issues that the researchers have not considered with regards to people with Parkinson’s?

The use of PwP and carers participants is well planned.
PwP/carers would be willing to take part as they can monitor changes and get advice on how to handle them and have assurance that their specialist/team are also being alerted/contactable. Testers should be aware that not all PwP have a carer.
However there is little mention of the Parkinson's specialist/team willingness to take part who are crucial for the app to be successful. Specialist/team are busy people and may not have the time to devote to being available. Funding for this application should be conditional on explicit support from specialist/team.
The testers have done as much as possible to make it easy for people to take part.
Testers should be aware that one NMS is apathy. So frequent checking on people's use is continuing after the first flush of interest/novelty to avoid drop out.

4. Have the researchers demonstrated Patient and Public Involvement (PPI) in shaping their research? (if applicable)

It is recognised that the best quality Parkinson's research is carried out when researchers work **with** patients and carers in the planning, delivery and/or dissemination of their research. This is called Patient and Public Involvement (PPI).

PPI can include, for example, people affected by Parkinson's working with researchers to identify the most relevant research questions, comment on the design of a study, develop recruitment and retention plans for participants, or offer advice as members of a project steering group.

- Do you think there are any strengths or weaknesses to how the research team have/ or plan to use PPI in their study? Do you have any suggestions for improvements?
- Do you think the resources and arrangements (such as travel/ subsistence expenses and staff time) allocated for PPI in their project are sufficient and appropriate; and do you think people would be willing to work with the researchers as a result?
- If the researchers have not done any PPI – do you have any recommendations for how people affected by Parkinson's could be involved in the study going forwards?

The researchers have shown high committment to PPI having a PwP and a carer as co-appplicants.

5. Do you have any additional comments about the application?

If you have any additional thoughts about the ways in which the application could be improved please add them below.

Avoid using "disease". One of the better applications using plain English with good list of abbreviations and glossary of terms. Even so some are missing eg IRAS and difference between qualitative and quantitatve. I recommend using consistent terms eg health care team and app.

Score

The listed characteristics are for guidance only. The characteristics are general statements on the overall quality of the application in each in funding category. They are not a checklist of minimum criteria for the funding category.

Funding category	Characteristics	Rating scale		
**Highly fundable**	• Clear and well written proposal.<br>• Very important research questions; likely to result in significant benefit for people affected by Parkinson's.<br>• Strong evidence of meaningful and well-planned patient and public involvement, with activities integrated at relevant points throughout the project.	Exceptional	10	☐
		Excellent quality research	9	☐
		Very good, bordering on excellent	8	☐
**Potentially fundable**	• Relevant research questions; likely to result in benefit for people affected by Parkinson's.<br>• All key aspects of application are clearly presented.<br>• Some evidence of patient and public involvement, with activities well planned and integrated at relevant points.	Good quality research	7	☐
		Above average quality research	6	☐
		Acceptable quality	5	☐
**Not fundable (without significant changes)**	• Research questions are not directly relevant to Parkinson's or are unlikely to result in benefit for people affected by Parkinson's.<br>• Key elements of the application are unclear.<br>• Limited evidence of patient and public involvement with unclear plans.	Borderline quality research	4	☐
		Below acceptable quality	3	☐
**Definitely not fundable**	• Irrelevant research questions.<br>• Unclear application.<br>• No or limited evidence of appropriate patient and public involvement in the research.	Many identified flaws	2	☐
		Serious weaknesses or major concerns	1	☐

Parkinson’s UK top 10 priority areas for improving everyday life	
For more information on the priority research areas and the full list, please visit our website.	
1	**Balance and falls**
2	**Stress and anxiety**
3	**Uncontrollable movements**
4	**Personalised treatments**
5	**Dementia**
6	**Mild thinking and memory problems**
7	**Monitoring symptoms**
8	**Sleep**
9	**Dexterity**
10	**Urinary problems**

Please tick here if you would be happy for us to use anonymised versions of your comments for future training and feedback purposes ☒

Completed review forms should be emailed to: researchapplications@parkinsons.org.uk

When reviewing project grant applications, forms should also be emailed to your Lay Review Co-ordinator.
